# Influence of PhoP and Intra-Species Variations on Virulence of *Yersinia pseudotuberculosis* during the Natural Oral Infection Route

**DOI:** 10.1371/journal.pone.0103541

**Published:** 2014-07-30

**Authors:** Fabio Pisano, Wiebke Heine, Maik Rosenheinrich, Janina Schweer, Aaron M. Nuss, Petra Dersch

**Affiliations:** Department of Molecular Infection Biology, Helmholtz Centre for Infection Research, Braunschweig, Germany; University of Louisville, United States of America

## Abstract

The two-component regulatory system PhoP/PhoQ has been shown to (i) control expression of virulence-associated traits, (ii) confer survival and growth within macrophages and (iii) play a role in *Yersinia* infections. However, the influence of PhoP on virulence varied greatly between different murine models of infection and its role in natural oral infections with frequently used representative isolates of *Y. pseudotuberculosis* was unknown. To address this issue, we constructed an isogenic set of *phoP* ^+^ and *phoP* ^−^ variants of strain IP32953 and YPIII and analyzed the impact of PhoP using *in vitro* functionality experiments and a murine oral infection model, whereby we tested for bacterial dissemination and influence on the host immune response. Our results revealed that PhoP has a low impact on virulence, lymphatic and systemic organ colonization, and on immune response modulation by IP32953 and YPIII, indicating that PhoP is not absolutely essential for oral infections but may be involved in fine-tuning the outcome. Our work further revealed certain strain-specific differences in virulence properties, which do not strongly rely on the function of PhoP, but affect tissue colonization, dissemination and/or persistence of the bacteria. Highlighted intra-species variations may provide a potential means to rapidly adjust to environmental changes inside and outside of the host.

## Introduction

The lifestyle of most enteric bacterial pathogens, particularly those frequently circulating between external reservoirs and warm-blooded hosts, demand efficient strategies to survey and respond to their permanently varying environments. Enteropathogenic bacteria are continuously confronted with rapid changes in nutrient and ion availability during their existence outside and within the host. In order to cope with this situation they evolved sophisticated sensory and signal transduction systems to monitor a large variety of chemical parameters and to convert them into intracellular signalling cascades to adjust their gene expression profile. The most rapid and efficient strategy of adjusting gene transcription involves the highly conserved two-component regulatory systems (TCSs). These TCSs typically comprise a membrane-spanning sensor kinase, which monitors extracellular components (e.g. certain nutrients or ions) and transduces the signal to a DNA-binding cognate response regulator via a histidine to aspartate phosphorelay [1]–[3].

Multiple TCSs have been identified in different enteric pathogens and many of them play an important role in the regulation of bacterial pathogenesis. Based on genome-wide *in silico* analysis it was predicted that the entero­pathogenic bacterium *Yersinia pseudotuberculosis* encodes 24 different TCSs [4], [5]. Several of these systems were found to promote tolerance towards certain environmental stresses (e.g. inorganic/organic acids/low pH, high salinity, low iron/magnesium, antimicrobial peptides such as polymyxin B, and hydrogen peroxide) and were shown to be important for virulence [4], .

One of the most important TCSs implicated in *Yersinia* virulence is the PhoP/PhoQ system. This system has been extensively characterized in *Yersinia* and related pathogens for its ability to sense low Mg^2+^ concentrations, low pH and the presence of cationic antimicrobial peptides, i.e. typical attributes of professional phagocytes [6]–[9]. These conditions are sensed by the sensor kinase PhoQ, which promotes phosphorylation and activation of the response regulator PhoP. The regulatory cascade elicited by PhoP confers the ability to survive and replicate within macrophages [6], [10]–. Furthermore, *phoP*-deficient *Y. pseudotuberculosis* mutants were found to be attenuated in different murine infection models, suggesting that growth within macrophages is an important feature of pathogenesis [6], [12]–[14].

*Y. pseudotuberculosis* is a zoonotic food-borne pathogen responsible for the onset of several gut-associated diseases (yersiniosis) ranging from self-limiting enteritis, enterocolitis, watery diarrhea to mesenterial lymphadenitis. In rare cases, the bacteria also induce autoimmune disorders such as reactive arthritis and other systemic infections [15]–[17]. *Y. pseudotuberculosis* is usually transmitted via the fecal-oral route. After ingestion, the bacteria travel to the ileum and cross the intestinal epithelial layer via M-cells overlaying the lymphoid follicles, known as Peyer’s patches. Within the lymphatic tissue, the bacteria are immediately confronted by resident and recruited phagocytes, mainly neutrophils, macrophages and dendritic cells (DCs) [18]. At this stage of the infection the bacteria prevent phagocytosis by these innate immune cells, which would otherwise destroy the bacteria once they are internalized. For this purpose, they use a type III secretion system (T3SS) to inject multiple effector proteins (the Yops and LcrV) into the phagocytes in order to disrupt the dynamics of the cell cytoskeleton, trigger apoptosis of macrophages and suppress the production of proinflammatory cytokines [19]. As a result, the pathogenic yersiniae are mainly found as aggregates of extracellular bacteria within abscesses or necrotic lesions where they effectively resist phagocytosis by neutrophils [20], [21]. Although the majority of bacteria appear to multiply in extracellular locations, there is also evidence that they can survive and replicate within macrophages. This process is likely to be important during the very early stages of the infection (<12 h). In a study by Fujimura *et al.* [22] *Y. pseudotuberculosis* was detected in rabbits within follicle-associated macrophages underneath M cells 4 h postinfection, and also the closely related *Yersinia* species *Y. pestis* and *Y. enterocolitica* have been identified within macrophages during the very early phases of an infection [23], [24]. Moreover, it was shown that all three pathogenic yersiniae are able to replicate in naïve and activated macrophages *in vitro* [25]–[28]. Based on this observation it was speculated that macrophages could provide a replicative niche in which the bacteria can (i) proliferate while protected from neutrophils that are recruited to the site of infection, (ii) avoid antigen presentation and therefore delay the development of a specific immune response, and (iii) be transported from the initial site of infection to deeper lymph tissues (Trojan horse model) [29]. Consequently, the ability to survive and replicate in macrophages would provide a significant selective advantage during pathogenesis.

Several studies have clearly demonstrated that the PhoP/PhoQ TCS is required for survival and replication of pathogenic yersiniae in macrophages and/or neutrophils [6], [12], [25], [29]–[31]. However, the role of the PhoP/PhoQ system and how the ability to replicate within macrophages affects the infection process in different *Yersinia* strains and species is less clear. Although, the PhoP-dependent ability to proliferate in phagocytes is conserved among different *Yersinia* species and strains, the defect in virulence observed with corresponding ortholog *phoP* mutants was very different. For instance, a strong attenuation (75-fold higher LD_50_) was reported for a *phoP* mutant of the *Y. pestis* strain GB, but only a modest defect in virulence was seen with a *phoP^−^* mutant of *Y. pestis* CO92 upon subcutaneous injections in mice (bubonic model) [12], [14]. In addition, PhoP does not seem to play a role for virulence of *Y. pestis* CO92 during aerosol infections of mice (pneumonic model) [14], whereas a much greater influence was observed after an aerosol challenge with *phoP*-deficient mutants of the *Y. pseudotuberculosis* strains IP32953 and IP2666 [13], [14]. Hence, the impact of a *phoP* mutation appears to depend on species and strain-specific differences that remodel regulatory networks and/or virulence-associated traits with different effects on their virulence phenotype.

One of the best characterized isolates of *Y. pseudotuberculosis*, the serotype O:3 strain YPIII, encodes a mutated and non-functional form of the *phoP* gene associated with an intracellular survival defect in macrophages and yet retains its ability to cause a lethal disease in the murine infection model [6], [32]. Due to the inactivating mutation of the *phoP* gene, the use of YPIII as a model pathogen for murine infections has often been criticized. However, an extensive analysis of the influence of *phoP* on the virulence properties of the YPIII strain is so far missing. In the present study, we took YPIII (*phoP^−^*) and the more virulent human isolate IP32953 (*phoP^+^*) as models of *Y. pseudotuberculosis* infections, constructed a YPIII *phoP^+^* and a IP32953 *phoP^−^* derivative, and determined the relevance of PhoP functionality both *in vitro* and *in vivo* during the natural oral infection route with particular interest on bacterial dissemination and modulation of the host immune response. Our results demonstrated that the impact of PhoP on the virulence of IP32953 and YPIII during oral infections is rather low. However, major strain-specific differences in tissue colonization and the induction of the innate and adaptive immune response were observed, indicating that different virulence properties do not mainly rely on the functionality of PhoP.

## Materials and Methods

### Ethics Statement

All animal experiments were performed in strict accordance with the German Recommendations of the Society of Laboratory Animal Science (GV-SOLAS) and the European Health Recommendations of the Federation of Laboratory Animal Science Associations (FELASA). The animal protocols were approved by the Niedersächsisches Landesamt für Verbraucherschutz und Lebensmit­telsicherheit: animal licensing committee permission no. 33.9.42502-04-12/1010. Animals were handled with appropriate care and all efforts were made to minimize suffering.

### Bacterial strains, cell culture, culture media and growth conditions

All bacterial strains used in this study are listed in Table **1**. If not stated differently, cultures of *E. coli* and *Y. pseudotuberculosis* were grown at 37°C and 25°C in LB (Luria-Bertani) broth, respectively. For bacterial selection, antibiotics were used in the following concentrations: chloramphenicol 30 µg/ml, kanamycin 50 µg/ml, carbenicilin 100 µg/ml. For Mg^2+^-dependent growth analysis of the *Y. pseudotuberculosis* strains, bacteria were grown in TMH medium at 25°C and 37°C [33], [34] without MgCl_2_ (referred to as low magnesium medium) or supplemented with 5 mM MgCl_2_.

**Table 1:** Bacterial strains, plasmids and primers.

Strain/Plasmid	Description	Reference
**Bacterial strains**		
*E. coli* K-12		
DH10β	F^−^ *endA1 recA1 galE15 galK16 nupG rpsL* Δ*lacX74* Φ80*lacZ*ΔM15*araD139Δ(ara, leu)7697 mcrAΔ(mrr-hsdRMS-mcrBC)*λ*^−^*	Invitrogen
S17–1 λpir	*recA thi pro hsdR^−^ M^+^(*RP4–2 Tc::Mu-Km::Tn7), *λpir*	
*Y. pseudotuberculosis*		
IP32953	human isolate, pYptb32953, serogroup O:1, *phoP* ^+^	[47]
YPIII	human isolate, pIB1, serogroup O:3, *phoP* ^M116H, R117st^°^p^	[48]
YPIP04	IP32953, Δ*phoPQ*, Kan^R^	This study
YPIP06	YPIP04, *phoPQ* ^YPIII^	This study
YP56	YPIII, Δ*phoPQ*, Kan^R^	[32]
YP149	YP56, *phoPQ* ^IP32953^	[32]
**Plasmids**		
pDM4	R6K derivate, *sacB^+^*, Cm^R^	[59]
pFU54	pSC101*-derived *rbs-luxCDABE* reporter plasmid, Cb^R^	[39]
pJNS02	pFU54 derivate, P*_cnfy_*::*luxCDABE,* Cb^R^	[32]
pVP3	pDM4 derivate, Kn^R^	This study
pWH1	pDM4 derivate, *phoPQ* ^YPIII^, Kn^R^	This study
pVP1	pDM4 derivate, *phoPQ* ^IP32953^, Kn^R^	[32]
**Primers**		
III925	5′-GCGCACTAGTGTCGTGGGTGCCAGCCG-3′	
III926	5′-GCGCGAGCTCCCAGCGGCGACGGCCTG-3′	
III927	5′-GCGCCTCGAGGTCGTGGGTGCCAGC-3′	
III964	5′-GCCAATGATAACCGTGGTAGTGC-3′	
III965	5′-TTTGCACTGTCAGATGGTGACGC-3′	
III966	5′-TCTCGACCACTTGGGGCGC-3′	
III967	5′-AAAGCCCTTAGGGGGAGCC-3′	

The corresponding restriction sites are underlined.

Murine J774A.1 macrophages were cultured in RPMI 1640 with GlutaMAX (Invitrogen) supplemented with 5% fetal calf serum (PAA Laboratories GmbH) at 37°C in the presence of 5% CO_2_.

### DNA manipulations and plasmid construction

All DNA manipulations, PCR, restriction digestions, ligations and transformations were performed using standard techniques as described previously [35], [36]. Plasmids used in the assays are shown in Table **1**. For the construction of a kanamycin resistance cassette with 500 bp flanking regions of the *phoPQ* locus on plasmid pVP3, the kanamycin resistance cassette from chromosomal DNA of YP56 was amplified (primer pair III925/III926) and ligated into the *Spe*I/*Sac*I sites of pDM4. The plasmid pWH1 was constructed by amplification of *phoPQ* of *Y. pseudotuberculosis* strain IP32953 with its 500 bp flanking regions using the primers III926 and III927. The fragment was ligated into the *Xho*I/*Sac*I sites of pDM4.

### Mutant strain constructions

In order to generate the *Y. pseudotuberculosis* IP32953 *phoPQ* knock-out mutant YPIP04, pVP3 was integrated into the *phoPQ* locus of IP32953 via conjugation as previously described [37]. Chromosomal integration of the fragment was selected by plating on *Yersinia* selective agar supplemented with kanamycin. Conjugants with an excision of the plasmid including the functional *phoPQ* operon of IP32953 were identified after plating on 10% sucrose and selection of fast-growing, carbenicillin sensitive strains. Absence of the *phoPQ* operon was tested by PCR. To construct *Y. pseudotuberculosis* IP32953 *phoP^−^* strain YPIP06 harbouring the non-functional *phoP* gene of YPIII, the plasmid pWH1 was conjugated into YPIP04. Chromosomal integration of the plasmid was selected by plating on *Yersinia* selective agar supplemented with chloramphenicol. The correct mutant was identified after plating on 10% sucrose as following: (i) fast-growing bacteria were isolated and tested for loss of the kanamycin cassette; (ii) presence of the *phoPQ* operon of YPIII was analyzed by PCR with primers III964, III965, III966, and III967; and (iii) presence of the *phoPQ* locus including the mutation of YPIII in *phoP* was confirmed by PCR and sequencing.

### Intracellular survival of *Y. pseudotuberculosis* in J774A.1 macrophages

Aliquots of 2×10^5^ J774A.1 macrophages were seeded into 24-well plates and activated with Phorbol-12-myristate-13-acetate (PMA) 48 h prior to infection. One hour before infection, the culture medium was replaced as previously described [38]. Overnight cultures of bacteria grown in LB medium at 25°C were washed with PBS and added to the J774A.1 macrophages in a multiplicity of infection (MOI) of 1 or 10. The number of living bacteria for infection was determined by plating on LB medium and assessment of colony forming units (CFU). The infected cells were incubated for 1 h at 37°C in an atmosphere consisting of 5% CO_2_ followed by a further 1 h incubation in medium supplemented with 8 µg/ml gentamicin. Bacterial uptake was calculated as the percentage of intracellular bacteria at t_0_ relative to the number of infecting bacteria. To assess bacterial uptake 2 h postinfection, cells were extensively washed with PBS, lysed with 0.1% Triton X-100 in PBS and lysates were plated on LB medium to determine CFU counts. At 8 h and 24 h postinfection, survival of bacteria was assessed as described previously [38]. For each strain, the proportion of bacterial uptake was determined by calculating the number of CFUs at uptake relative to total number of CFUs used for infection. Survival rates were calculated by the number of CFUs at 8 and 24 h relative to the number of CFUs at uptake, respectively. Statistical analysis was performed using the student’s t-test.

### Infection of mice

BALB/c female mice aged between 10–12 weeks were purchased from Janvier (Le Genest Saint Isle, St Berthevin Cedex, France) and housed in individually ventilated cages on a 12 h light: dark cycle under specific pathogen-free conditions according to the FELASA recommendations in the animal facility of the Helmholtz Centre for Infection Research, Braunschweig, Germany. Animals were given food and water ad libitum; 14 hours prior to infection food was removed and made available again after the infection. *Y. pseudotuberculosis* strains used for infections were grown over night in LB medium at 25°C, washed and resuspended in PBS. For survival assays, groups of five weight-matched mice were infected intragastrically using a gavage needle with approximately 5×10^8^ CFUs of *Y. pseudotuberculosis* YPIII, IP32953, YPIII *phoP* ^+^ and IP32953 *phoP* ^−^. The infected mice were monitored every day for 14 days to determine the survival rate, body weight and health status. Survival experiments were performed in triplicates. As mice lost more than 20% of their original body weight they were sacrificed by CO_2_ asphyxiation. Statistical analysis was performed using the Log-rank (Mantel-Cox) test.

For CFU assessment in infected organs, groups of 5 weight-matched mice were orally infected with approximately 2×10^8^ CFUs of *Y. pseudotuberculosis phoP^+^* or *phoP^−^* variants of strains YPIII and IP32953 in three independent experiments. At day three postinfection, mice were sacrificed by CO_2_ asphyxiation. Peyer’s patches (PPs), mesenteric lymph nodes (MLNs), spleen and liver were excised aseptically. Subsequently, all organs were weighed and homogenized in sterile PBS at 15,000 rpm for 30 seconds using a Polytron PT 2100 homogenizer (Kinematica, Switzerland). Bacterial organ burden was determined by plating serial dilutions of the homogenates on *Yersinia* selective agar (Oxoid). The CFUs were counted 48 h after plating and are given as the number of CFUs per gram of organ tissue. The data was statistically analyzed using One-way ANOVA followed by Tukey’s multiple comparisons test.

### Immune response analysis

Groups of 5 weight-matched mice were challenged intragastrically with 2×10^8^ bacteria of *Y. pseudotuberculosis* YPIII, IP32953, YPIII *phoP* ^+^ or IP32953 *phoP* ^−^, in four independent experiments. Groups of 5 uninfected mice in three independent experiments were included as controls. At day 3 postinfection, the infected and the uninfected mice were euthanized via CO_2_ asphyxiation. Subsequently, PPs, MLNs and spleen were isolated and single cell suspensions were generated in PBS supplemented with 0.2% BSA by meshing the organs through a cell strainer (100 µm, BD). To eliminate the erythrocytes, splenocytes were incubated in ACK-buffer (150 mM NH_4_Cl, 10 mM KHCO_3_, 1 mM EDTA, pH 7.4) for 3 min. Cell suspensions of all analyzed tissues were counted using a Beckman Coulter Counter. Subsequently, 1×10^6^ or 2.5×10^6^ cells were stained. Staining was performed using following antibodies/chemicals: I) Live/Dead Fixable Blue Dead Cell Stain (Invitrogen), CD16/CD32 (Biolegend, cl.93), CD19:FITC (eBioscience, cl.N418), CD3:V450 (BD Bioscience, cl.17A2), NKp46:APC (Biolegend, cl.29A1.4); II) Live/Dead Fixable Blue Dead Cell Stain (Invitrogen), CD16/CD32 (Biolegend, cl.93), CD3e:Biotin (BD Biosciences, cl.145-2C11), CD19:Biotin (eBioscience, cl.eBio1D3), panNK:Biotin (Biolegend, cl.DX5), Streptavidin:FITC (BD Bioscience), CD11b:eFluor450 (eBioscience, cl.M1/70), CD45R:PerCP-Cy5.5 (eBioscience, cl.RA3-6B2), F4/80:PE (eBioscience, cl.BM8), Ly6G:PE-Cy7 (BD Bioscience, cl.1A8), Ly6C:APC (Biolegend, cl.HK1.4), CD11c:APC-eFluor780 (eBioscience, cl.N418). Data were collected with FACS LSR-Fortessa (BD Biosciences), and analyzed with FlowJo (Treestar). Cells that were F4/80^hi^ were considered as macrophages, CD11c^hi^ Ly6G^−^ cells as conventional dendritic cells (cDC), Ly6G^+^ cells as neutrophils, Ly6C^+^ CD45R^+^ cells as plasmacytoid dendritic cells (pDC), CD11b^+^ cells as monocytes. Moreover, CD19^+^ cells were considered as B cells, NKp46^+^ cells as natural killer cells and CD3^+^ cells as T cells. Controls were performed both as single stainings and as FMOs (fluorescence minus one). The gating strategy for B cells, T cells and NK cells are shown in Fig. **S1**, and for macrophages, cDCs, pDCs, neutrophils, monocytes in Fig. **S2**. Living cell numbers were statistically analyzed using One-way ANOVA with Tukey’s multiple comparisons test.

### Cytokine/chemokine multiplex assay

Heart blood was taken from 8 weight-matched mice either infected with *Y. pseudotuberculosis* YPIII, IP32953, YPIII *phoP* ^+^ or IP32953 *phoP* ^−^ at 3 days post infection and from 5 uninfected mice. Samples were obtained from three independent experiments and the serum was separated from the cellular part by two-step centrifugation at 5,000 rpm for 8 min after clotting of the blood. The serum was analyzed using the MILLIPLEX Mouse cytokine/chemokine panel (Merck Millipore). Cytokine levels were statistically analyzed by using One-way ANOVA with Tukey’s post hoc test.

### Luciferase assay

*Y. pseudotuberculosis* YPIII and YPIII *phoP^+^* (YP149) carrying either pJNS02 [32] or the empty vector pFU54 [39] were inoculated 1∶50 from overnight cultures and grown in LB medium with antibiotics at 25°C. Every 2 h, samples were taken and analyzed for luciferase activity and optical density (OD) at 600 nm in technical duplicates with Varioskan flash (Thermo scientific). Relative luciferase units were normalized to the corresponding OD_600_.

## Results

### Comparative analysis of *Y. pseudotuberculosis* YPIII and IP32953 *phoP* ^+^ and *phoP* ^−^ variants

Previous work demonstrated that the PhoP/PhoQ TCS affects replication of *Y. pseudotuberculosis* in macrophages, but its role in virulence, in particular upon the natural oral infection route is not clear. Over the last decades, mainly *Y. pseudotuberculosis* isolates YPIII (serotype O:3) and IP32953 (serotype O:1b) were used to elucidate *Y. pseudotuberculosis* pathogenicity. However, it was recently found that YPIII carries a mutation in the PhoP-encoding gene YPK_1715, leading to a non-functional version of PhoP [6]. Although it has been reported that PhoP/PhoQ is required for virulence in *Y. pseudotuberculosis* 32777 by the intragastric route [6], strain YPIII is still able to cause lethal disease in the oral murine mouse infection model [40].

**Figure 1:**
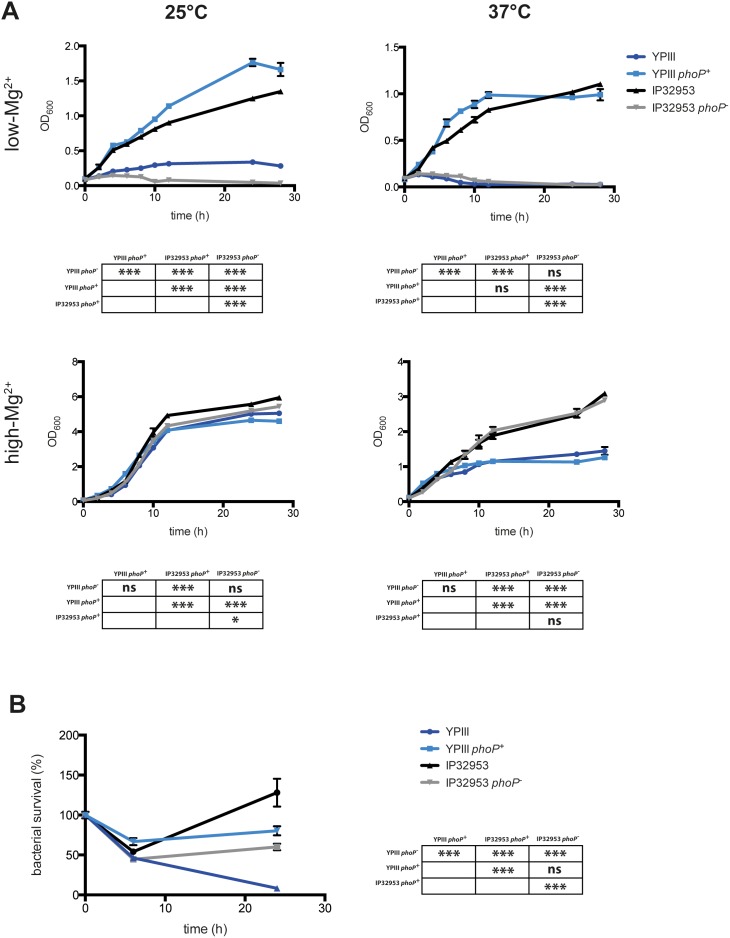
Loss of PhoP in *Y. pseudotuberculosis* YPIII and IP32953 impairs growth in a low magnesium environment and survival inside J774A.1 macrophages. (A) *Y. pseudotuberculosis* strains YPIII (*phoP* ^−^), YP149 (YPIII *phoP^+^*), IP32953 (*phoP* ^+^) and YPIP06 (IP32953 *phoP* ^−^) were inoculated into TMH medium with either low or high magnesium concentrations (5 mM) and grown at either 25°C or 37°C. The graph represents one independent experiment out of three replicates. Statistical analysis was performed using Two-way ANOVA with Tukey's multiple comparisons test at 24 h of growth (*, p<0.05; ***p<0.001). (B) Activated J774A.1 macrophages were infected with *Y. pseudotuberculosis* strains YPIII (*phoP* ^−^), YP149 (YPIII *phoP^+^*), IP32953 (*phoP* ^+^) and YPIP06 (IP32953 *phoP* ^−^) at an MOI 1 for 1 h. The infection was stopped with gentamicin and bacterial uptake was determined 2 h postinfection. Bacterial survival inside the macrophages was determined over time. The graph represents a summary of three independent experiments with four replicates respectively. Statistical analysis was performed using Two-way ANOVA with Tukey's multiple comparisons test at 24 h post assessment of bacterial uptake (***, p<0.001). Error bars represent SEM.

In order to test the role of PhoP in *Y. pseudotuberculosis* and compare its impact between the representative isolates YPIII and IP32953, we first constructed an isogenic *phoP* ^+^ and *phoP* ^−^ derivative of both strains: YP149 (YPIII *phoP* ^+^_IP32953_) and YPIP06 (IP32953 *phoP* ^−^_YPIII_) (for further details see Material and Methods), tested their growth in low and high Mg^2+^ environments, and determined their ability to replicate within macrophages (Fig. **1**). In accordance with previous studies [6], only YPIII *phoP^+^* and IP32953 were able to grow under low Mg^2+^ concentrations, while YPIII and IP32953 *phoP^−^* were both growth defective at 25°C and 37°C, and supplementation with 5 mM MgCl_2_ enabled growth of all tested strains (Fig. **1A**). Bacterial uptake inside macrophages was similar among the four tested strains, and corresponded to approximately 40% at MOI 1 and 20% at MOI 10 of the inoculated bacteria, respectively (data not shown). As expected, YPIII was clearly defective for replication in macrophages (Fig. **1B**), whereas its *phoP^+^* derivative displayed significantly increased survival, replicating during the first day of infection despite the presence of the virulence plasmid. A similar reduction in survival and replication in macrophages due to the loss of *phoP* was observed for the *Y. pseudotuberculosis* strain IP32953 (Fig. **1B**). However, the bacterial counts of IP32953 were considerably higher compared to YPIII *phoP^+^*, indicating that IP32953 is much less sensitive to killing by the macrophage phagosome. Taken together, these results are in general agreement with previously published studies [6], and confirm the correct replacement of the *phoP* alleles.

### PhoP influence on virulence of *Y. pseudotuberculosis* YPIII and IP32953

**Figure 2:**
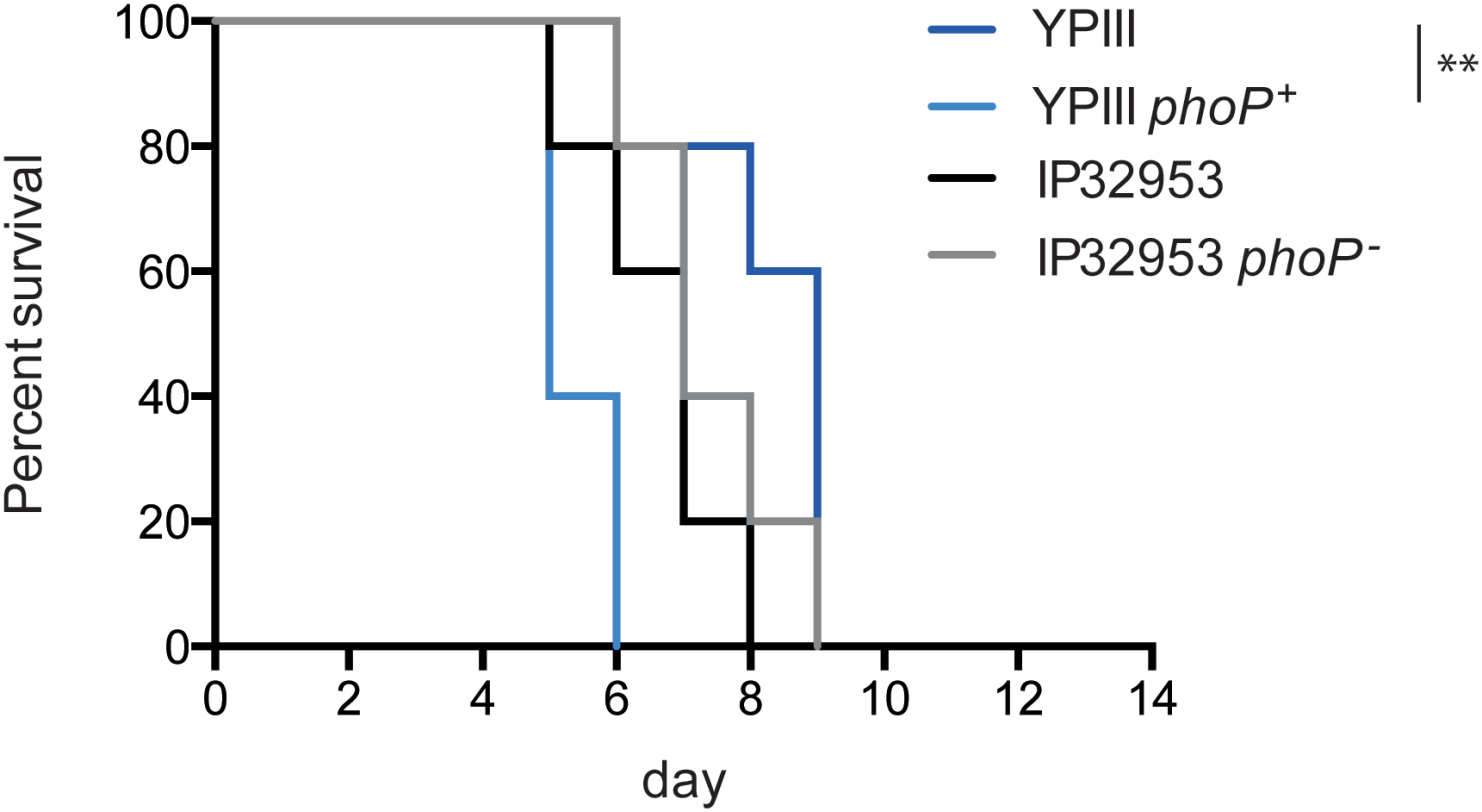
PhoP influences virulence properties of *Y. pseudotuberculosis*. BALB/c mice were infected with 5×10^8^ CFU of *Y. pseudotuberculosis* strains YPIII (*phoP* ^−^) (n = 15), YP149 (YPIII *phoP^+^*) (n = 15), IP32953 (*phoP* ^+^) (n = 15) and YPIP06 (IP32953 *phoP* ^−^) (n = 15). Survival and fitness of five mice per group were recorded for 14 d. The survival curves were compared with the log-rank test (**, p<0.01).

It was previously shown that the loss of a functional *phoPQ* locus results in the increased survival of mice orally infected with *Y. pseudotuberculosis* serogroup O:1 strain 32777 (100-fold less virulent estimated by LD_50_) [6]. To better understand the role of PhoP and to determine the impact of the non-functional *phoP* ^−^_YPIII_ allele on virulence of *Y. pseudotuberculosis*, mice were orally infected with approximately 5×10^8^ CFU of *phoP^+^* and *phoP^−^* variants of YPIII and IP32953. Presence of a functional PhoP resulted only in a mild increase in lethality for the YPIII *phoP^+^* strain in comparison to YPIII, whereas statistical comparison of the survival curves did not highlight any PhoP-dependent difference between the IP32953 *phoP^+^* and *phoP^−^* variants (Fig. **2**).

**Figure 3:**
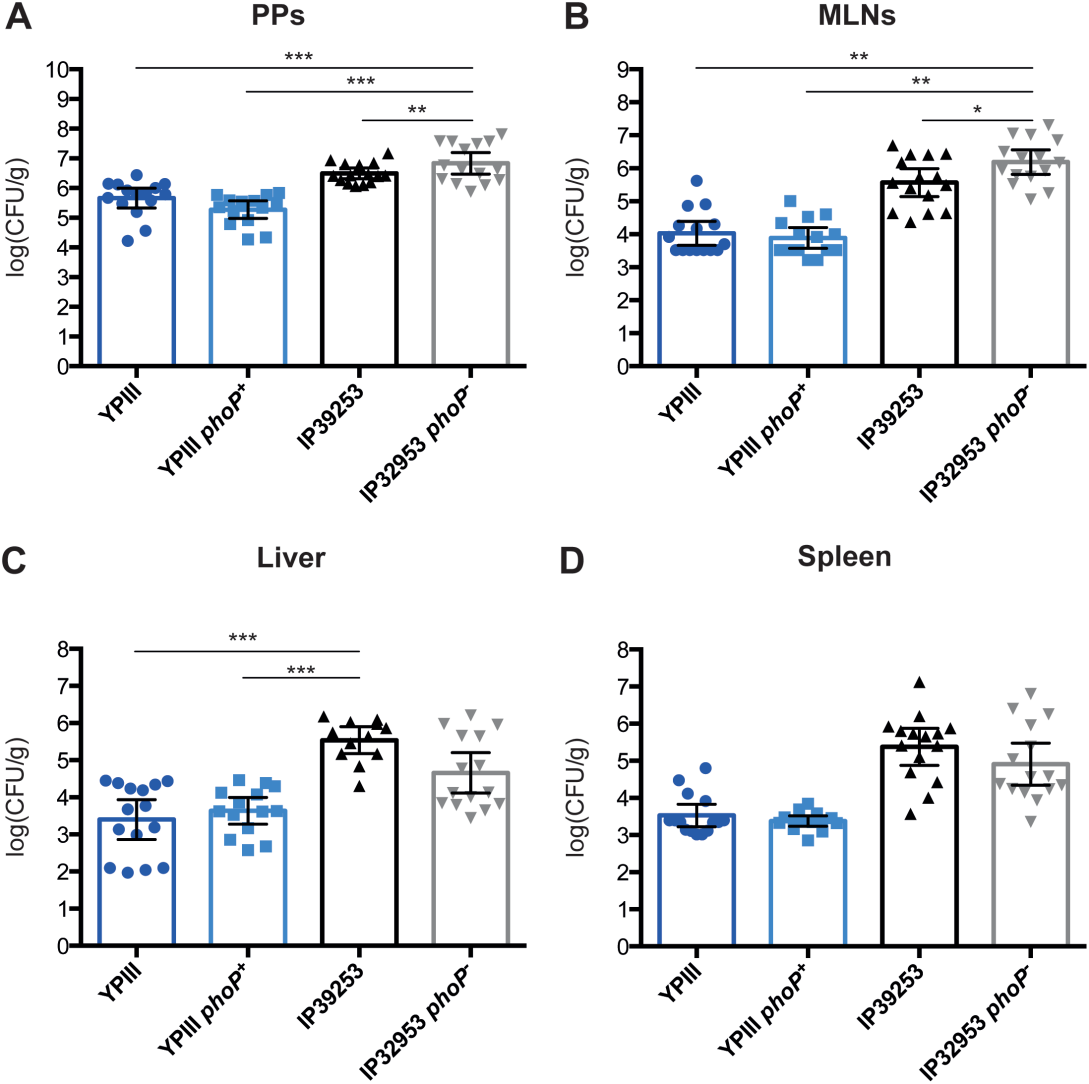
Colonization of *Y. pseudotuberculosis* YPIII (*phoP* ^−^), YP149 (YPIII *phoP^+^*), IP32953 (*phoP* ^+^) and YPIP06 (IP32953 *phoP* ^−^) in host tissues. BALB/c mice were challenged with 2×10^8^ CFU of *Y. pseudotuberculosis* strains YPIII (*phoP* ^−^) (n = 15), YP149 (YPIII *phoP^+^*) (n = 15), IP32953 (*phoP* ^+^) (n = 15) and YPIP06 (IP32953 *phoP* ^−^) (n = 15). At day 3 postinfection mice were sacrificed. Numbers of bacteria cells from the different strains were determined in the lymphatic organs PPs (A), MLNs (B) and the systemic organs liver (C) and spleen (D). Data from four independent experiments were pooled. Bacterial loads were compared using One-way ANOVA with Tukey’s post hoc test (*, p<0.05; **, p<0.01; ***, p<0.001).

Upon oral infection, *Y. pseudotuberculosis* disseminates from the PPs and/or small intestinal epithelium to the MLNs and systemic organs like the liver and spleen. To explain the observed virulence properties in the oral infection model, we investigated the influence of PhoP on colonization and dissemination of YPIII and IP32953 in more detail. For each strain, mice were orally challenged with 2×10^8^ CFUs. At day 3 postinfection, mice were sacrificed and the bacterial burden in the PPs, MLNs, spleen and liver was assessed (Fig. **3**). Analysis of the organ burden revealed significant strain specific differences in the colonization and dissemination ability. IP32953 and IP32953 *phoP^−^* populated all assessed organs with higher efficiency than YPIII and YPIII *phoP^+^* (Fig. **3**). Furthermore, the YPIII and YPIII *phoP^+^* strains disseminated less efficiently to the MLNs, liver and spleen (Fig. **3**). Despite the small differences observed for the survival of the mice (Fig. **2**), organ colonization by YPIII was not affected by the presence of a functional copy of *phoP*. Indeed, YPIII *phoP^+^* and YPIII *phoP^−^* bacteria were found at similar levels in all analyzed organs. In contrast, the lymphatic tissues (PPs and MLNs) were populated slightly more efficiently by IP32953 in the absence of a functional PhoP (Fig. **3A, B**), while less bacteria were isolated from the liver and spleen (Fig. **3C, D**). In summary, this data indicates that PhoP appears to be dispensable for host tissue colonization by YPIII, and only minor changes were observed for IP32953.

### PhoP is not important for induction of the immune response

**Figure 4:**
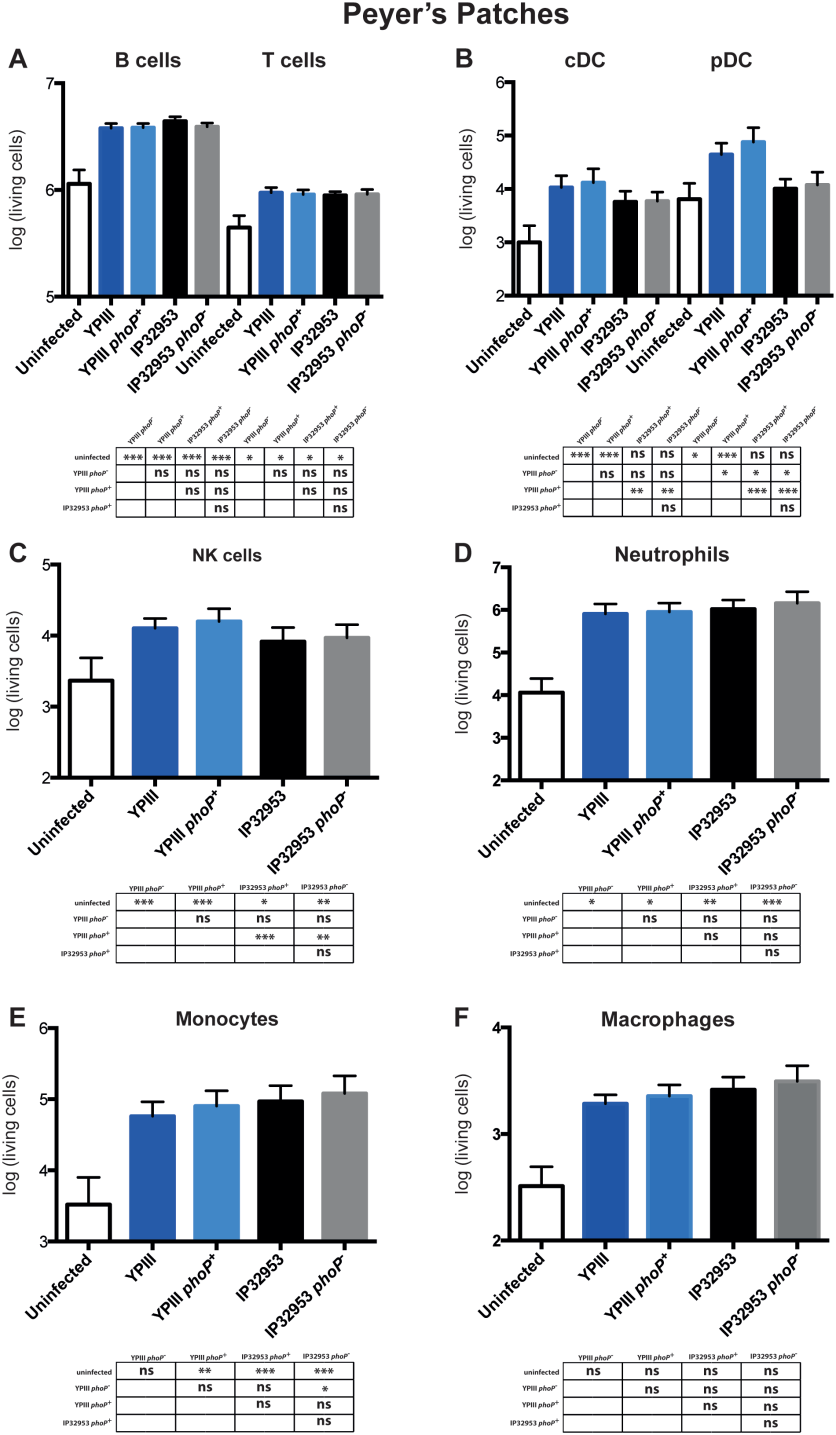
Immune response analysis in the PPs of BALB/c mice induced by *phoP* ^+^ and *phoP* ^−^ derivatives of *Y. pseudotuberculosis* YPIII or IP32953. Mice were challenged with 2×10^8^ CFU of *Y. pseudotuberculosis* strains YPIII (*phoP* ^−^) (n = 20), YP149 (YPIII *phoP^+^*) (n = 20), IP32953 (*phoP* ^+^) (n = 20) and YPIP06 (IP32953 *phoP* ^−^) (n = 20). A control group of uninfected mice (n = 20) was included. At day three postinfection, living cell numbers of B cells and T cells (A), plasmacytoid and conventional DCs (B), NK cells (C), neutrophils (D), monocytes (E) and macrophages (F) in the PPs were analyzed. The data represents the total cell number per organ in a logarithmic scale. Data from four independent experiments were pooled. Statistical analysis was performed using One-way ANOVA with Tukey’s post hoc test (*, p<0.05; **, p<0.01; ***, p<0.001).

Many studies have shown that cells of the innate immunity like macrophages and neutrophils, and adaptive immunity like CD4^+^ T_h_1 and CD8^+^ T cells are crucial in the control of *Yersinia* infections [41]–[44]. In 2009, Kumar *et al*., showed that a virulence-plasmid cured *Y. pseudotuberculosis* Δ*phoPQ* mutant is diminished in the T_h_1-type immune response in a murine systemic infection model following intravenous inoculation [45]. However, a deeper dissection of the PhoP-driven immune response in mice infected by the natural intragastric route is so far missing. To this purpose, the influence of the response regulator PhoP and strain-driven differences on innate and adaptive immune responses to *Y. pseudotuberculosis* infection were assessed. Three days after oral infection, mice were sacrificed to analyze the cell type composition of the PPs, MLNs and spleen. To this purpose, living cell levels of natural killer cells (NK cells), neutrophils, monocytes, dendritic cells (DCs), macrophages, B cells and T cells were evaluated (Fig. **4** **–** **6**; **Fig. S3–S5**).

**Figure 5:**
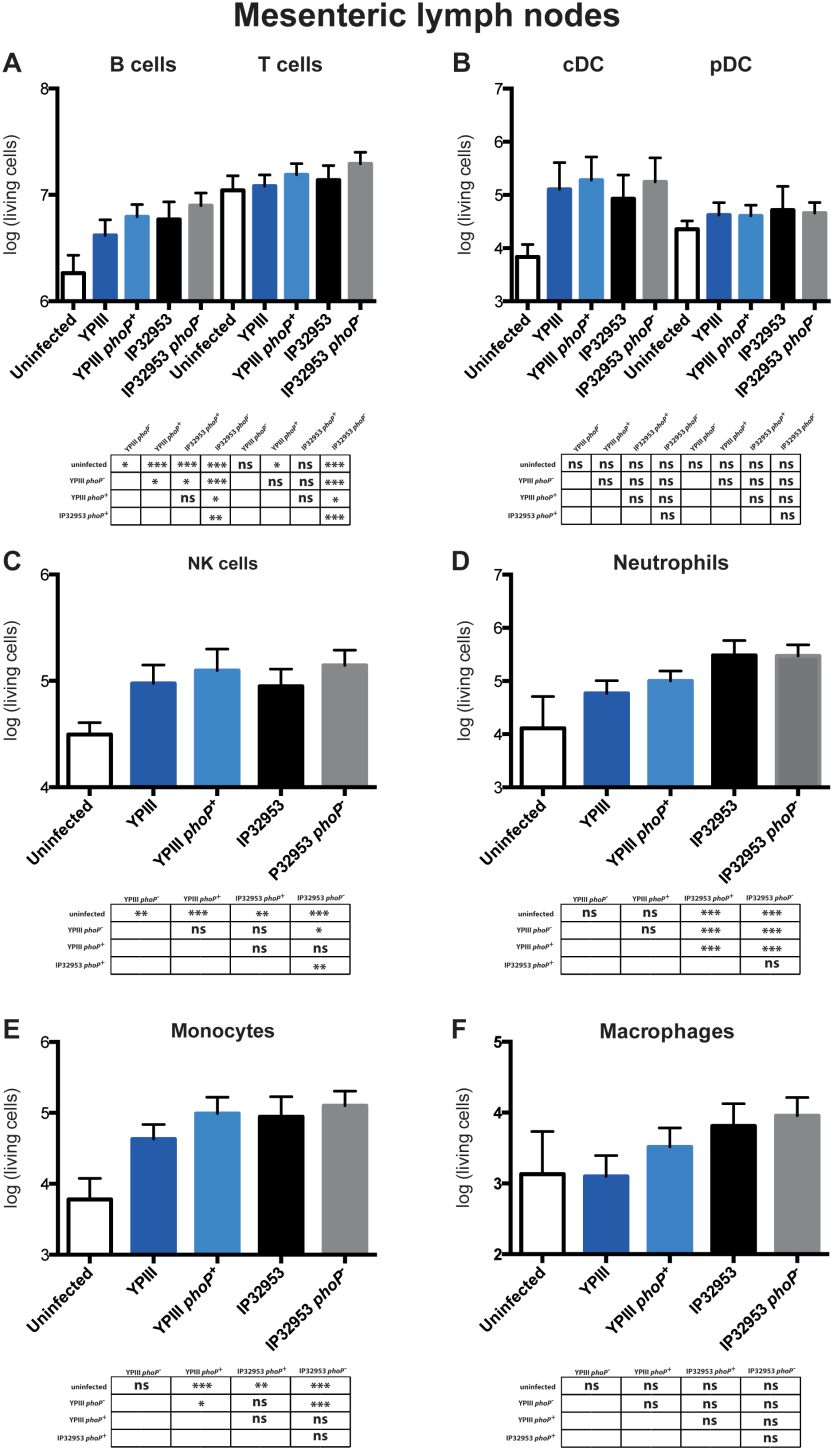
Immune response analysis in the mesenteric lymph nodes of BALB/c mice infected with *phoP* ^+^ and *phoP* ^−^ derivatives of *Y. pseudotuberculosis* YPIII or IP32953. Mice were challenged with 2×10^8^ CFU of *Y. pseudotuberculosis* strains YPIII (*phoP* ^−^) (n = 20), YP149 (YPIII *phoP^+^*) (n = 20), IP32953 (*phoP* ^+^) (n = 20) and YPIP06 (IP32953 *phoP* ^−^) (n = 20); a control group of uninfected mice (n = 15) was included. At day three postinfection living cell numbers of B cells and T cells (A), plasmacytoid and conventional DCs (B), NK cells (C), neutrophils (D), monocytes (E) and macrophages (F) in the MLNs were analysed. The data represents the total cell number per organ in a logarithmic scale. Data from four independent experiments were pooled. Living cell numbers were statistically analyzed using One-way ANOVA with Tukey’s post hoc test (*, p<0.05; **, p<0.01; ***, p<0.001).

**Figure 6:**
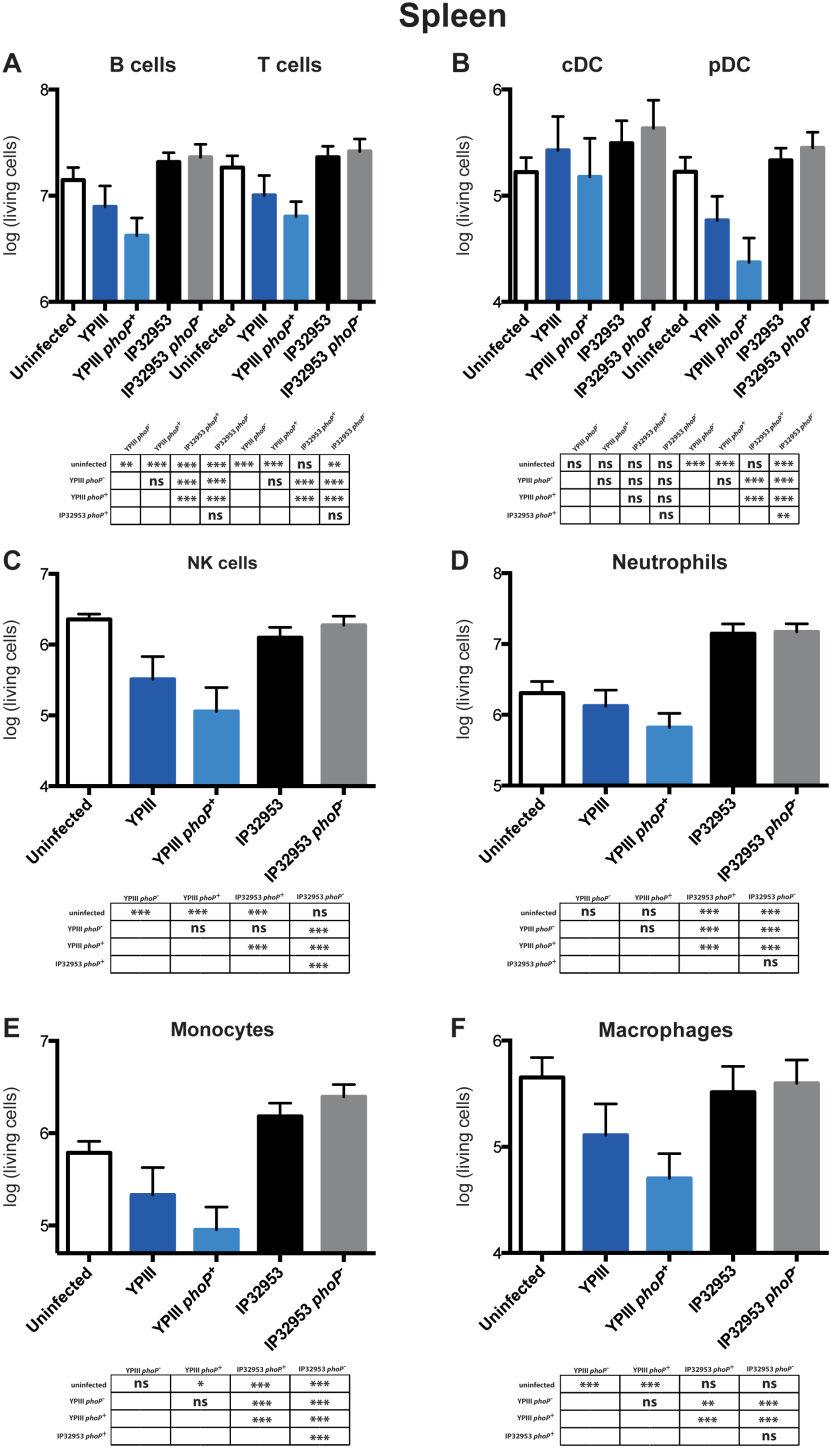
Immune response analysis in the spleen of BALB/c mice infected with *phoP* ^+^ and *phoP* ^−^ derivatives of *Y. pseudotuberculosis* YPIII or IP32953. Mice were challenged with 2×10^8^ CFU of *Y. pseudotuberculosis* strains YPIII (*phoP* ^−^) (n = 20), YP149 (YPIII *phoP^+^*) (n = 20), IP32953 (*phoP* ^+^) (n = 20) and YPIP06 (IP32953 *phoP* ^−^) (n = 20); a control group of uninfected mice (n = 15) was included. At day three postinfection living cell numbers of B cells and T cells (A), plasmacytoid DCs and conventional DCs (B), NK cells (C), neutro­phils (D), monocytes (E) and macrophages (F) in the spleen were analyzed. The data represents the total cell number per organ in a logarithmic scale. Data from four independent experiments were pooled. Living cell numbers were compared using One-way ANOVA with Tukey’s post hoc test (*, p<0.05; **, p<0.01; ***, p<0.001).

In the PPs and MLNs of *Y. pseudotuberculosis* infected mice, a significant infiltration/increase of innate and adaptive immune cells was observed (Fig. **4** **,** **5**). In particular, neutrophils, monocytes and NK cells increased substantially and to a similar extent within the lymphoid tissues in YPIII and IP32953 infected mice (Fig. **4C, D, E** **,** Fig. **5C, D, E**). Only pDC levels did not raise in the PPs of IP32953 infected mice (Fig. **4B**), irrespective of the functionality of PhoP. Analysis of the mesenteric lymph node composition highlighted a stronger increase in the relative abundance of macrophages and neutrophils in mice infected with the strains IP32953 and IP32953 *phoP^−^* in comparison to YPIII and YPIII *phoP^+^* infected ones (Fig. **S4**). Overall, the presence of PhoP had no, or only a very small effect on the infiltration of immune cells into the gut-associated lymphatic tissues.

Similarly in the spleen, no significant difference in the levels of cells of the adaptive and innate immune systems were observed during infection with *Y. pseudotuberculosis* strains IP32953 and IP32953 *phoP^−^* (Fig. **6**). However, YPIII and YPIII *phoP^+^* induced a significant decrease (likely due to a cytotoxic effect) in most of the immune cells, including NK cells, pDCs, macrophages and monocytes, splenic B cells and T cells. Thereby, YPIII seems to influence exclusively the number of pDCs and not cDCs (Fig. **6B**). Furthermore, reduction/killing of immune cells was enhanced in the *phoP* ^+^ derivative of YPIII (Fig. **6**). Notably, spleens of mice infected with the IP32953 derived strains displayed a higher percentage of neutrophils and monocytes in comparison to the YPIII infected mice (Fig. **S5**). Taken together, these results show profound differences in the modulation of the immune response elicited by YPIII and IP32953, whereby only YPIII leads to a PhoP-dependent reduction of immune cells in the spleen during intragastric infection.

### PhoP influence on the production of proinflammatory cytokines

The inflammatory process responsible for immune cell recruitment and function is largely mediated by cytokines that are produced upon infection [46]. Therefore, we further analyzed the levels of 32 different cytokines in sera of mice infected with YPIII, YPIII *phoP^+^*, IP32953 and IP32953 *phoP^−^* as well as uninfected mice via a cytokine multiplex assay.

**Figure 7:**
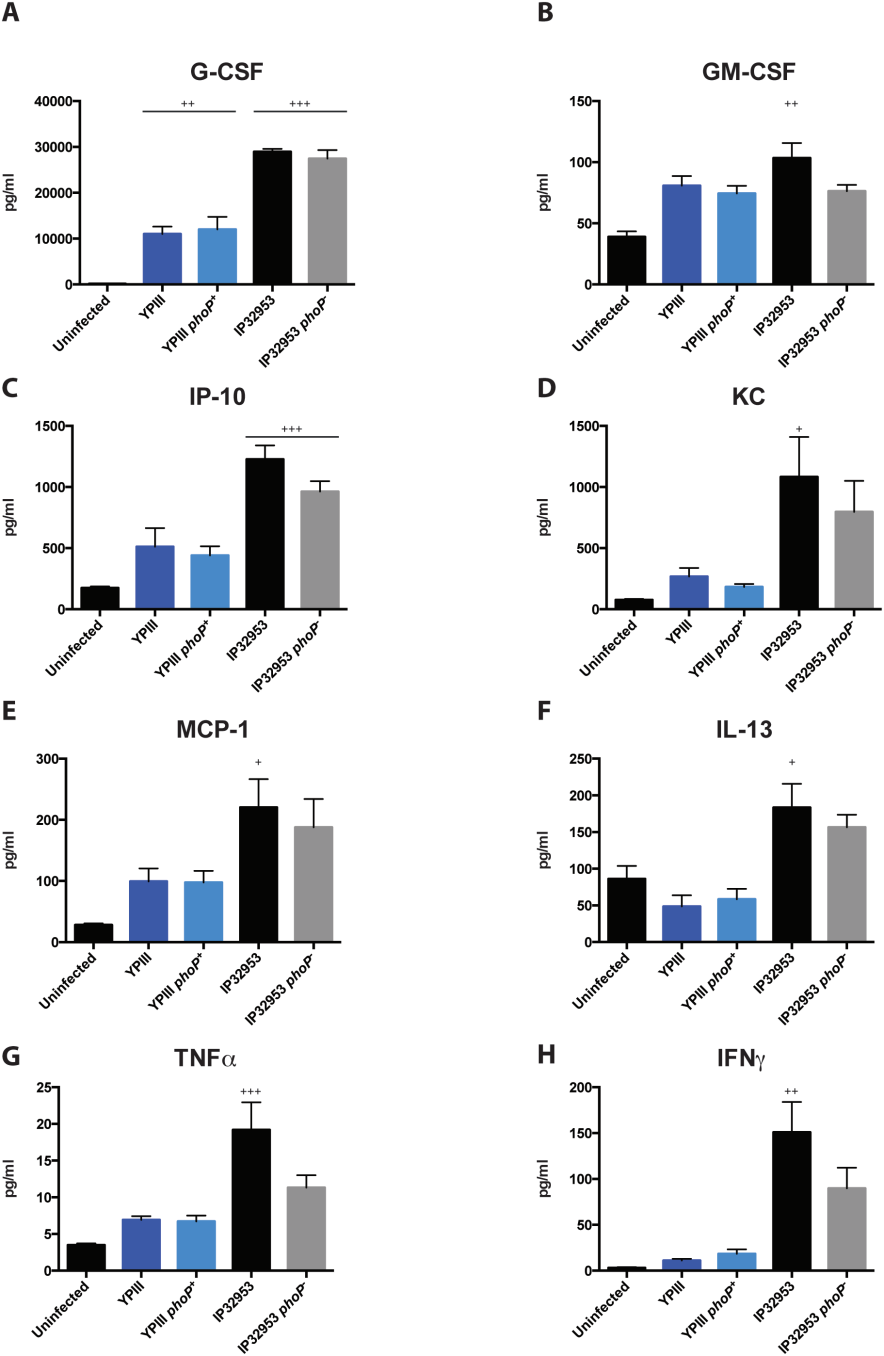
Cytokine analysis of the serum of BALB/c mice infected with *phoP* ^+^ and *phoP* ^−^ derivatives of *Y. pseudotuberculosis* YPIII or IP32953. Mice were challenged with 2×10^8^ CFU of *Y. pseudotuberculosis* strains YPIII (*phoP* ^−^) (n = 8), YP149 (YPIII *phoP^+^*) (n = 8), IP32953 (*phoP* ^+^) (n = 8) and YPIP06 (IP32953 *phoP* ^−^) (n = 8) in three independent experiments; a control group of uninfected mice (n = 5) was included. At day three postinfection blood was isolated to determine the cytokine profile of the serum. Cytokine levels were statistically analyzed using One-way ANOVA with Tukey’s post hoc test (+, p<0.05; ++, p<0.01; +++, p<0.001). + indicate a comparison to the uninfected group.

Infection with *Yersinia* induced only an increase of selected cytokines at the systemic level with major differences between the different serotypes (Fig. **7**). Notably, a significantly higher production of these cytokines was observed in IP32953 infected mice compared to mice challenged with YPIII regardless of the functionality of *phoP*. Among the most induced cytokines was G-CSF, which is responsible for the mobilization and activation of hematopoietic cells from the bone marrow (Fig. **7A**), and the neutrophil-, monocyte- and DC-chemoattractant cytokines MCP-1, IP-10 and KC (Fig. **7C–E**). Concentrations of these cytokines were 2–4 fold higher in the blood of IP32953-infected mice compared to YPIII-infected mice. Most likely, this inter-serotype difference in cytokine production is reflected in different neutrophil population levels within the infected organs (Fig. **4** **–** **6**). Similarly, serum levels of the pleiotropic cytokine IFNγ and the potent systemic inflammation inducer TNFα were only slightly increased in mice infected with YPIII and YPIII *phoP^+^* whereas significantly higher amounts of IFNγ were produced in response to IP32953 and IP32953 *phoP^−^*. Notably, lack of PhoP in the IP32953 strain also resulted in a considerably lower TNFα, IFNγ and IP-10 response in comparison to the wild-type strain. Finally, the serum analysis also highlighted a strain-dependent induction of IL-13. This cytokine is responsible for the establishment of a T_h_2 immune response against extracellular pathogens. Infection with the YPIII-derived strains did not induce a systemic increase of IL-13 in comparison to uninfected mice, while a 2-fold rise in its levels was observed in mice infected with IP32953 and IP32953 *phoP^−^*. In contrast, levels of the potent macrophage activator cytokine GM-CSF were only slightly affected by both *Y. pseudotuberculosis* strains at the time-point after infection (Fig. **7B**). Taken together, these results reveal major differences in the systemic cytokine response between mice infected with YPIII and IP32953, mostly in a PhoP-independent fashion.

### PhoP induces expression of CNF_Y_ in *Y. pseudotuberculosis* YPIII

**Figure 8:**
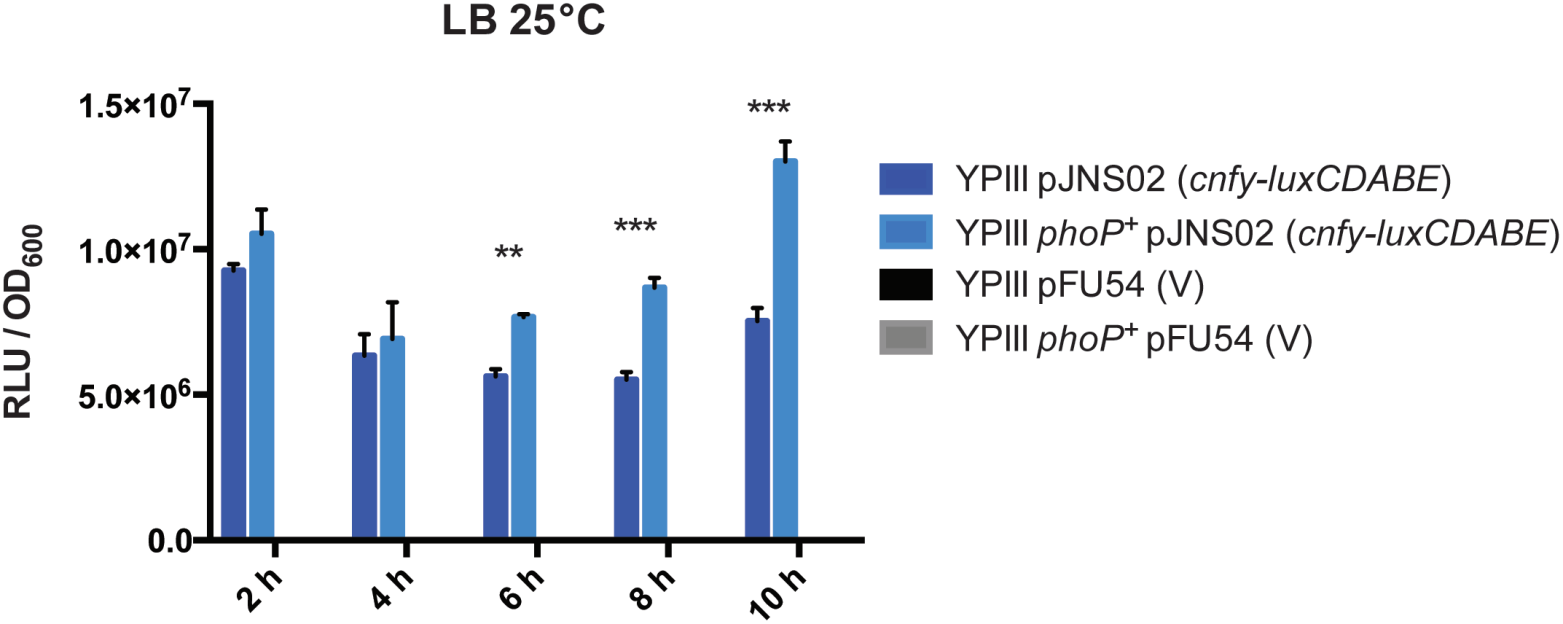
PhoP increases expression of *cnfY* in *Y. pseudotuberculosis* YPIII. *Y. pseudotuberculosis* strain YPIII and the isogenic YPIII *phoP^+^* variant carrying a *luxCDABE*-reporter plasmid fused to the promoter of *cnfY* (pJNS02) or the empty vector (pFU54) were grown in LB medium. The *luxCDABE*-reporter activity and OD_600_ was measured over time. The figure represents three independent experiments done in triplicate. Statistical analysis of the RLU/OD_600_ was performed with the student’s t-test (**, p<0.01; ***, p<0.001).

Immune cell composition revealed a substantial increase in the capability of YPIII *phoP^+^* strain to influence the distribution of cells of the innate immune system in the spleen in comparison to the wild-type strain YPIII (Fig. **6**). Given the similar titres of bacteria in this organ (Fig. **3**), the mechanism seems to be independent of the quantity of bacterial cells. We recently identified the cytotoxic necrotizing factor of *Yersinia* (CNF_Y_) as a major player in determining the depletion of immune cells in the spleen via the improvement of Yop delivery in the cytoplasm of host cells [32]. Therefore, we investigated whether the presence of PhoP was able to affect *cnfY* expression *in vitro*. YPIII and YPIII *phoP^+^* carrying a plasmid with the *cnfY* promoter fused to the luciferase operon *luxCDABE* were grown from exponential to stationary phase in LB medium and the growth and bioluminescence measured. Expression of the reporter fusion was significantly higher in the YPIII *phoP^+^* strain compared to YPIII after exponential phase, indicating that PhoP activates *cnfY* expression upon entry into the stationary phase (Fig. **8**). These results suggest a role for PhoP on CNF_Y_ expression during the course of infection, and may account for the increased cytotoxic effect of the YPIII *phoP^+^* strain in comparison to the parent strain YPIII. We further investigated whether presence of CNF_Y_ had an influence on the systemic cytokine response (Fig. **S6**). However, only small changes between YPIII and an isogenic *cnfY* mutant were observed, indicating that major differences in IFN-**γ**, TNF-**α**, MCP-1, KC, IL-13 and IP-10 secretion between mice infected with YPIII and IP32953 are not caused by CNF_Y_.

## Discussion

Since the discovery of a mutation in the *phoP* gene of the *Y. pseudotuberculosis* isolate YPIII, leading to a truncated PhoP protein, and given the central role of the PhoP/PhoQ TCS in the ability to survive and replicate in macrophages *in vitro* in all human pathogenic *Yersinia* species [6], [12], [25], [29]–[31], several concerns about the employment of YPIII as a representative example for *Y. pseudotuberculosis* in murine infection models were raised. Yet, the PhoP-deficient YPIII strain is still highly virulent and maintains its lethality during oral mouse infections [32], in contrast to a *phoP* mutant derivative of *Y. pseudotuberculosis* isolate 32777 [6]. Overall, a very different impact of PhoP on *Yersinia* virulence was reported [12]–[14], suggesting that the importance of PhoP for the control of crucial pathogenicity factors differs significantly among strains of the three different human pathogenic *Yersinia* species. However, a direct comparison of the outcome of the infection is difficult as distinct experimental settings (e.g. different infection routes, doses, and mouse strains) were employed.

To overcome this problem, we studied the contribution of PhoP to virulence of the best-characterized *Y. pseudotuberculosis* strains YPIII and IP32953 during the natural oral infection route. Both strains were and are still frequently used to analyze *Y. pseudotuberculosis* pathogenicity. The serotype 1b strain IP32953, a close evolutionary relative to *Y. pestis*, is a highly virulent clinical isolate from a human patient, which efficiently colonizes lymphatic tissues and organs after oral uptake and causes a high lethality of mice [47]. YPIII is a very well characterized mouse virulent isolate that induces severe acute inflammatory responses and the formation of necrotic areas in infected tissues [48]. Different to most other strains, YPIII expresses the cytotoxic necrotizing factor CNF_Y_ which plays a dominant role in the progression of disease and a deletion of the corresponding *cnfY* gene was shown to abrogate virulence [32]. Interestingly, the *cnfY* gene is truncated and thus is non-functional in most *Y. pestis* and *Y. pseudotuberculosis* strains, including IP32953 [49]. The present study was designed to dissect the relevance of PhoP upon oral infection with YPIII and IP32953 in terms of bacterial colonization/dissemination, and with respect to the elicited immune response.

In the present study, we show that loss of the response regulator has only a small effect on the lethality of YPIII, and no effect on IP32953. Dispensability of PhoP is further reflected in similar colonization levels (Fig. **3**). No (YPIII) or only very small variations (IP32953) of the bacterial loads were observed in the different infected tissues. Such differences between the IP32953-derived strains may indicate that absence of PhoP results in a slight reduction of the dissemination of IP32953 from the lymphoid tissues (PPs and MLNs) to the systemic organs (liver and spleen). PhoP expression in three different *Y. pseudotuberculosis* isolates, including YPIII, was shown to repress biofilm formation in a *Caenorhabditis elegans* model [50]. However, based on our results this property does not seem to be important for tissue colonization and dissemination of the bacteria in mammals. This hypothesis is supported by the fact that other *Y. pseudotuberculosis* mutants defective in biofilm formation (e.g. Δ*hmsT*) were found to be fully virulent in a murine oral infection model [50].

The analysis of the host immune response during a YPIII and IP32953 infection further demonstrated that PhoP has no or very little effect on the composition of immune cells in the infected tissues. A dominant influence was only found in the spleen infected with YPIII, but not with IP32953. YPIII infection alone caused a strong decrease of several immune cell populations, in particular of the professional phagocytes. In a previous study we showed that the CNF_Y_ toxin secreted by YPIII is responsible for the reduction of the immune cells and causes an enormous and unique splenotoxic effect in comparison to IP32953 (Δ*cnfY*) [32]. It is unclear whether this effect is due to a long-range delivery of the toxin to the spleen from the gut compartment or is locally induced. However, it seems that this effect is PhoP-dependent since the reduction of the immune cell populations is more pronounced in mice infected with the YPIII *phoP^+^* strain. This hypothesis was confirmed by expression analyses demonstrating a stronger induction of *cnfY* transcription in the presence of a functional *phoP* copy.

Interestingly, pDC distribution differed in a strain-dependent, but PhoP-independent fashion. In mice infected with the strains IP32953 and IP32953 *phoP^−^* the number of pDC remained almost unaltered in all analyzed organs. However, in mice infected with YPIII and YPIII *phoP* ^+^ we observed a decrease in the number of pDC in the spleen and a corresponding increase of this cell type in the Peyer’s patches. This suggests that in response to YPIII infection pDC may migrate from the spleen to the Peyer’s patches. We additionally observed that MLNs of mice infected with IP32953 strains were predominantly populated by neutrophils and macrophages and the spleens by neutrophils and monocytes, while a similar pattern was not observed in YPIII infected mice. However, this inter-strain difference might rather be due to the higher colonization of spleen and MLN achieved by the IP32953 strain than to a specific immune response (Fig. **S4**, **S5**).

Analysis of the systemic production of cytokines, however, highlighted PhoP-dependent differences in the IP32953-triggered immune response. Significantly lower levels of several cytokines, in particular IFNγ and TNFα, were detected in mice infected with the *phoP*-deficient mutant derivative. One could hypothesize that this difference is due to a different PhoP-dependent behaviour of the bacteria inside phagocytes. However, considering that intracellular *Y. pseudotuberculosis* are rare [51], and that the numbers of phagocytes remains unaffected in the IP32953 infected tissues (Fig. **4** **–** **6**), it is more likely that the observed increase may be due to the higher amounts of wild-type bacteria in the liver and spleen (Fig. **3**).

A second striking finding from our studies is that YPIII colonizes the infected tissues and/or disseminates much less efficiently to the PPs, the MLNs, liver and spleen than IP32953. Similarly, IP32953 was found to disseminate more rapidly in a murine model of lung infections compared to IP2666, a *Y. pseudotuberculosis* isolate that also expresses the CNF_Y_ toxin and robustly colonizes lungs, but only sporadically disseminates to the spleen and liver [52]. The different dissemination capacity might also be reflected in the profound PhoP-independent differences observed in the immune response to YPIII and IP32953. Especially the population of professional phagocytes in the infected tissues and systemic production of cytokines varied significantly during oral infections with the two virulent isolates. The basis for these differences is still unclear. However, expression and function of important adhesins and adhesin regulators were found to be very different between the two *Y. pseudotuberculosis* strains [53], [54] (A. M. Nuss, unpublished results). In particular, expression of invasin seems to be much more induced in IP32953 compared to YPIII [54]. We further identified variations between the YadA molecules of both isolates. Interestingly, several amino acids are changed or deleted in YadA_IP32953_ compared to YadA_YPIII_ clustered in a region of the YadA head domain previously shown to be crucial for autoagglutination, fibronectin binding and host cell invasion [55]. These and/or other differences in the function and expression profile of adhesin molecules may account for the variances in dissemination. Furthermore, different adhesin properties could change: (i) resistance of the host complement; (ii) efficiency of Yop translocation into professional phagocytes and (iii) specificity of immune cell types translocated with the Yops. These assumptions are supported by a recent study demonstrating that the adhesins as well as host serum factors drive Yop translocation into specific immune cell types [54]. Alternatively, other pathogenicity factors, i.e. the CNF_Y_ of YPIII shown to enhance Yop translocation into the phagocytes and NK cells [32] may contribute to the differences in the colonization efficiency and the immune response observed between YPIII and IP32953. Considering the role of the Yop proteins in suppression of the host immune response, including inhibition of phagocytic uptake, induction of apoptosis of professional phagocytes, alterations of cytokine production and hindrance of immune cell recruitment [19], [56], a contribution of CNF_Y_ to the strain-specific differences seems very likely. Therefore, we favor the idea that the strain-dependent phenotype in the murine infection model observed in this study relies upon different pathogenic mechanisms between the strains rather than on the presence of a functional PhoP.

The overall, more virulent infection by IP32953 reflected by faster killing of the host correlates with higher bacterial loads in the infected tissues and the higher proinflammatory cytokine responses in infected animals. In contrast, the slow dissemination of strain YPIII associated with an overall low induction of the host immune response may be advantageous for long-term colonization of the intestinal tract. Due to the powerful and long-range effect of the CNF_Y_ toxin compared to the local and equally strong effect of Yops, it could also be hypothesized that loss of PhoP function in YPIII might be even more advantageous for long-term colonization of the host, as its absence extends the lifetime of the host without a significant reduction of the bacterial load in the infected tissues. Furthermore, reduced expression of CNF_Y_ in the absence of PhoP is still sufficient to stimulate Yop translocation into professional phagocytes to block uptake, but lower toxin concentrations seems to reduce rapid killing of the immune cells, which would result in a less severe form of tissue destruction and slower killing of the host compared the *phoP* ^+^ derivative of YPIII.

In summary, findings in this and other recent studies highlight important strain-specific differences in the virulence properties of *Y. pseudotuberculosis* despite the fact that the genetic variability within this species is generally limited [52], [54], [57], [58]. The three mouse-virulent strains YPIII, IP32953 and IP2666, which are frequently used for the analysis of *Y. pseudotuberculosis* pathogenicity vary in their individual virulence factors, YPIII (e.g. *phoP* ^−^), IP2666 (e.g. *psaE* ^−^), IP32953 (e.g. *cnfY* ^−^), and in the expression level of important virulence regulators and virulence-associated traits. This may explain why controversial virulence phenotypes were often reported. Combined, these observations raise questions regarding the validity and significance of infection studies using a single strain as a species representative. Nonetheless, the analysis of the pathogenicity of a particular representative virulent *Y. pseudotuberculosis* isolate has and will continue to yield further insights into the structure, function and regulation of virulence factors and the induced immune responses. However, we pose that a more comparative analysis of multiple representatives of a particular *Yersinia* species will be a prerequisite to separate general/core virulence strategies from individual or species-specific adaptation strategies to certain mammalian hosts or host niches, an approach which is particularly important to develop successful diagnostic tools and anti-infectives.

## Supporting Information

Figure S1 **Gating strategy for T cells, B cells and NK cells.** The collected flow cytometry data was compensated and analyzed with FlowJo. At first, cells were gated for living cells by plotting Live Dead versus FSC-A followed by a double doublet-exclusion (FSC-A against FSC-H; SSC-H against SSC-W). The remaining singlets were gated for leukocytes by plotting FSC-A against SSC-A. At next, the auto fluorescent cells (AF^+^) were excluded (auto-fluorescence (AF) versus Live Dead). The remaining autofluorescence negative (AF^−^) leukocytes were gated for CD19^+^ cells (B cells). Subsequently, the CD19^−^ cells were gated for CD3^+^ cells (T cells) and NKp46^+^ (NK cells). Cell proportions of living T cells, B cells and NK cells were calculated by the frequency of living cells.
(PDF)

Figure S2 **Gating strategy for cDCs, pDCs, neutrophils, macrophages and monocytes.** The collected flow cytometry data was compensated and analyzed with FlowJo. At first, cells were gated for living cells by plotting Live Dead versus FSC-A followed by a double doublet-exclusion (FSC-A against FSC-H; SSC-H against SSC-W). The remaining singlets were gated for leukocytes by plotting FCS-A against SSC-A. At next, the leukocytes were gated for CD3/CD19/panNK lineage negative cells (Lin^−^) by plotting AF against CD3/CD19/panNK. The Lin^−^ cells were gated for F4/80^hi^ cells (macrophages) by plotting F4/80 against Ly6G. Subsequently, the F4/80^hi^ negative cells were gated against auto-fluorescence and SSC-A to exclude AF^+^ cells. Afterwards, the AF^−^ cells were gated for CD11c^hi^ cells (cDCs) by plotting Ly6G against CD11c. The CD11c^hi^ negative cells were gated against CD45R and Ly6G for Ly6G^+^ cells (neutrophils). There after, the Ly6G^−^ cells were plotted against CD11b and Ly6C for CD11b^+^ cells (monocytes). Finally, The CD11b^−^ cells were gated for CD45R^+^Ly6C^+^ cells (pDCs). Cell proportions of living macrophages, cDCs, neutrophils, monocytes and pDCs were calculated by the frequency of living cells.
(PDF)

Figure S3 **Immune response analysis in the PPs of BALB/c mice induced by** ***phoP*** **^+^ or** ***phoP*** **^−^ derivates of** ***Y. pseudotuberculosis*** **YPIII or IP32953.** Mice were challenged with 2×10^8^ CFU of *Y. pseudotuberculosis* strains YPIII (*phoP* ^−^) (n = 20), YP149 (YPIII *phoP* ^+^) (n = 20), IP32953 (*phoP* ^+^) (n = 20) and YPIP06 (IP32953 *phoP* ^−^) (n = 20). A control group of uninfected mice (n = 15) was included. At day three postinfection proportions of living B cells and T cells (A), cDCs and pDCs (B), NK cells (C), neutrophils (D), monocytes (E), macrophages (F) in the PPs were analyzed. Data from four independent experiments were pooled. Population percentages were analyzed using One-way ANOVA with Tukey’s post hoc test (*, p<0.05; **, p<0.01; ***, p<0.001).
(PDF)

Figure S4 **Immune response analysis in the MLNs of BALB/c mice induced by** ***phoP*** **^+^ or** ***phoP*** **^−^ derivates of** ***Y. pseudotuberculosis*** **YPIII or IP32953.** Mice were challenged with 2×10^8^ CFU of *Y. pseudotuberculosis* strains YPIII (*phoP* ^−^) (n = 20), YP149 (YPIII *phoP* ^+^) (n = 20), IP32953 (*phoP* ^+^) (n = 20) and YPIP06 (IP32953 *phoP* ^−^) (n = 20). A control group of uninfected mice (n = 15) was included. At day three postinfection proportions of living B cells and T cells (A), cDCs and pDCs (B), NK cells (C), neutrophils (D), monocytes (E), macrophages (F) in the MLNs were analyzed. Data from four independent experiments were pooled. Population percentages were analyzed using One-way ANOVA with Tukey’s post hoc test (*, p<0.05; **, p<0.01; ***, p<0.001).
(PDF)

Figure S5 **Immune response analysis in the spleen of BALB/c mice induced by** ***phoP*** **^+^ or** ***phoP*** **^−^ derivates of** ***Y. pseudotuberculosis*** **YPIII or IP32953.** Mice were challenged with 2×10^8^ CFU of *Y. pseudotuberculosis* strains YPIII (*phoP* ^−^) (n = 20), YP149 (YPIII *phoP* ^+^) (n = 20), IP32953 (*phoP* ^+^) (n = 20) and YPIP06 (IP32953 *phoP* ^−^) (n = 20). A control group of uninfected mice (n = 15) was included. At day three postinfection proportions of living B cells and T cells (A), cDCs and pDCs (B), NK cells (C), neutrophils (D), monocytes (E), macrophages (F) in the spleen were analyzed. Data from four independent experiments were pooled. Population percentages were analyzed using One-way ANOVA with Tukey’s post hoc test (*, p<0.05; **, p<0.01; ***, p<0.001).
(PDF)

Figure S6 **Cytokine analysis of the serum of BALB/c mice infected with** ***Y. pseudotuberculosis*** **YPIII or YP147.** Mice were challenged with 2×10^8^ CFU of *Y. pseudotuberculosis* strains YPIII (*phoP* ^−^) (n = 8) or YP147 (YPIII *phoP* ^−^ Δ*cnfY*) (n = 8); a control group of uninfected mice (n = 5) was included. At day three postinfection blood was isolated to determine the cytokine profile of the serum. G-CSF (**A**), GM-CSF (**B**), IP-10 (**C**), KC (**D**), MCP-1 (**E**), IL-13 (**F**), TNFα (**G**), IFNγ (**H**). Cytokine levels were statistically analysed using One-way ANOVA with Tukey’s post hoc test (+, p<0.05; ++, p<0.01; +++, p<0.001). + indicate a comparison to the uninfected group.
(PDF)

## References

[1] Beier D, Gross R (2006) Regulation of bacterial virulence by two-component systems. Curr Opin Microbiol 9: 143–152. 16481212 10.1016/j.mib.2006.01.005

[2] Robinson VL, Buckler DR, Stock AM (2000) A tale of two components: a novel kinase and a regulatory switch. Nature Structural Biology 7: 626–633. 10932244 10.1038/77915

[3] West AH, Stock AM (2001) Histidine kinases and response regulator proteins in two-component signaling systems. Trends in Biochemical Sciences 26: 369–376. 11406410 10.1016/s0968-0004(01)01852-7

[4] Flamez C, Ricard I, Arafah S, Simonet M, Marceau M (2008) Phenotypic analysis of *Yersinia pseudotuberculosis* 32777 response regulator mutants: new insights into two-component system regulon plasticity in bacteria. Int J Med Microbiol 298: 193–207. 17765656 10.1016/j.ijmm.2007.05.005

[5] Marceau M (2005) Transcriptional regulation in *Yersinia*: an update. Current Issues in Molecular Biology 7: 151–177. 16053248

[6] Grabenstein JP, Marceau M, Pujol C, Simonet M, Bliska JB (2004) The response regulator PhoP of *Yersinia pseudotuberculosis* is important for replication in macrophages and for virulence. Infect Immun 72: 4973–4984. 15321989 10.1128/IAI.72.9.4973-4984.2004 PMC517447

[7] Garcia Vescovi E, Soncini FC, Groisman EA (1996) Mg2+ as an extracellular signal: environmental regulation of *Salmonella* virulence. Cell 84: 165–174. 8548821 10.1016/s0092-8674(00)81003-x

[8] Lejona S, Aguirre A, Cabeza ML, Garcia Vescovi E, Soncini FC (2003) Molecular characterization of the Mg2+-responsive PhoP-PhoQ regulon in *Salmonella enterica* . Journal of Bacteriology 185: 6287–6294. 14563863 10.1128/JB.185.21.6287-6294.2003 PMC219391

[9] Perez JC, Shin D, Zwir I, Latifi T, Hadley TJ, et al (2009) Evolution of a bacterial regulon controlling virulence and Mg(2+) homeostasis. PLoS Genet 5: e1000428. 19300486 10.1371/journal.pgen.1000428 PMC2650801

[10] Groisman EA, Mouslim C (2006) Sensing by bacterial regulatory systems in host and non-host environments. Nature Reviews Microbiology 4: 705–709. 16894339 10.1038/nrmicro1478

[11] Groisman EA (2001) The pleiotropic two-component regulatory system PhoP-PhoQ. Journal of Bacteriology 183: 1835–1842. 11222580 10.1128/JB.183.6.1835-1842.2001 PMC95077

[12] Oyston PC, Dorrell N, Williams K, Li SR, Green M, et al (2000) The response regulator PhoP is important for survival under conditions of macrophage-induced stress and virulence in *Yersinia pestis* . Infect Immun 68: 3419–3425. 10816493 10.1128/iai.68.6.3419-3425.2000 PMC97616

[13] Fisher ML, Castillo C, Mecsas J (2007) Intranasal inoculation of mice with *Yersinia pseudotuberculosis* causes a lethal lung infection that is dependent on *Yersinia* outer proteins and PhoP. Infect Immun 75: 429–442. 17074849 10.1128/IAI.01287-06 PMC1828392

[14] Bozue J, Mou S, Moody KL, Cote CK, Trevino S, et al (2011) The role of the *phoPQ* operon in the pathogenesis of the fully virulent CO92 strain of *Yersinia pestis* and the IP32953 strain of *Yersinia pseudotuberculosis* . Microb Pathog 50: 314–321. 21320584 10.1016/j.micpath.2011.02.005

[15] Galindo CL, Rosenzweig JA, Kirtley ML, Chopra AK (2011) Pathogenesis of *Y. enterocolitica* and *Y. pseudotuberculosis* in human yersiniosis. J Pathog 2011: 182051. 22567322 10.4061/2011/182051 PMC3335670

[16] Koornhof HJ, Smego RA Jr, Nicol M (1999) Yersiniosis. II: The pathogenesis of *Yersinia* infections. Eur J Clin Microbiol Infect Dis 18: 87–112. 10219574 10.1007/s100960050237

[17] Smego RA, Frean J, Koornhof HJ (1999) Yersiniosis I: Microbiological and clinicoepidemiological aspects of plague and non-plague *Yersinia* infections. Eur J Clin Microbiol Infect Dis 18: 1–15. 10192708 10.1007/s100960050219

[18] Wershil BK, Furuta GT (2008) 4. Gastrointestinal mucosal immunity. Journal of Allergy and Clinical Immunology 121: S380–383; quiz S415. 10.1016/j.jaci.2007.10.023 18241686

[19] Viboud GI, Bliska JB (2005) *Yersinia* outer proteins: role in modulation of host cell signaling responses and pathogenesis. Annu Rev Microbiol 59: 69–89. 15847602 10.1146/annurev.micro.59.030804.121320

[20] Simonet M, Richard S, Berche P (1990) Electron microscopic evidence for *in vivo* extracellular localization of *Yersinia pseudotuberculosis* harboring the pYV plasmid. Infect Immun 58: 841–845. 2307522 10.1128/iai.58.3.841-845.1990 PMC258544

[21] Hanski C, Kutschka U, Schmoranzer HP, Naumann M, Stallmach A, et al (1989) Immunohistochemical and electron microscopic study of interaction of *Yersinia enterocolitica* serotype O8 with intestinal mucosa during experimental enteritis. Infect Immun 57: 673–678. 2917779 10.1128/iai.57.3.673-678.1989 PMC313160

[22] Fujimura Y, Kihara T, Mine H (1992) Membranous cells as a portal of *Yersinia pseudotuberculosis* entry into rabbit ileum. J Clin Electron Microsc 25: 35–45.

[23] Une T (1977) Studies on the pathogenicity of *Yersinia enterocolitica*. I. Experimental infection in rabbits. Microbiol Immunol 21: 341–363. 909458

[24] Finegold MJ (1969) Pneumonic plague in monkeys. An electron microscopic study. American Journal of Pathology 54: 167–185. 4974722 PMC2013466

[25] Pujol C, Bliska JB (2003) The ability to replicate in macrophages is conserved between *Yersinia pestis* and *Yersinia pseudotuberculosis* . Infect Immun 71: 5892–5899. 14500510 10.1128/IAI.71.10.5892-5899.2003 PMC201058

[26] Brzostek K, Raczkowska A, Zasada A (2003) The osmotic regulator OmpR is involved in the response of *Yersinia enterocolitica* O:9 to environmental stresses and survival within macrophages. FEMS Microbiol Lett 228: 265–271. 14638433 10.1016/S0378-1097(03)00779-1

[27] Yamamoto T, Hanawa T, Ogata S, Kamiya S (1996) Identification and characterization of the *Yersinia enterocolitica* gsrA gene, which protectively responds to intracellular stress induced by macrophage phagocytosis and to extracellular environmental stress. Infection and Immunity 64: 2980–2987. 8757824 10.1128/iai.64.8.2980-2987.1996 PMC174178

[28] Pujol C, Grabenstein JP, Perry RD, Bliska JB (2005) Replication of *Yersinia pestis* in interferon gamma-activated macrophages requires *ripA*, a gene encoded in the pigmentation locus. Proc Natl Acad Sci U S A 102: 12909–12914. 16120681 10.1073/pnas.0502849102 PMC1200267

[29] Pujol C, Bliska JB (2005) Turning *Yersinia* pathogenesis outside in: subversion of macrophage function by intracellular yersiniae. Clin Immunol 114: 216–226. 15721832 10.1016/j.clim.2004.07.013

[30] Grabenstein JP, Fukuto HS, Palmer LE, Bliska JB (2006) Characterization of phagosome trafficking and identification of PhoP-regulated genes important for survival of *Yersinia pestis* in macrophages. Infect Immun 74: 3727–3741. 16790745 10.1128/IAI.00255-06 PMC1489716

[31] O'Loughlin JL, Spinner JL, Minnich SA, Kobayashi SD (2010) *Yersinia pestis* two-component gene regulatory systems promote survival in human neutrophils. Infect Immun 78: 773–782. 19933831 10.1128/IAI.00718-09 PMC2812203

[32] Schweer J, Kulkarni D, Kochut A, Pezoldt J, Pisano F, et al (2013) The cytotoxic necrotizing factor of *Yersinia pseudotuberculosis* (CNFY) enhances inflammation and Yop delivery during infection by activation of Rho GTPases. PLoS Pathog 9: e1003746. 24244167 10.1371/journal.ppat.1003746 PMC3820761

[33] Higuchi K, Carlin CE (1957) Studies on the nutrition and physiology of *Pasteurella pestis*. I. A casein hydrolyzate medium for the growth of *Pasteurella pestis* . Journal of Bacteriology 73: 122–129. 13405873 10.1128/jb.73.1.122-129.1957 PMC289757

[34] Straley SC, Bowmer WS (1986) Virulence genes regulated at the transcriptional level by Ca2+ in *Yersinia pestis* include structural genes for outer membrane proteins. Infection and Immunity 51: 445–454. 3002984 10.1128/iai.51.2.445-454.1986 PMC262351

[35] Sambrook J (2001) Molecular Cloning: A Laboratory Manual,: Cold Spring Harbor Laboratories, Cold Spring Harbor, NY.

[36] Miller JH (1992) A short course in bacterial genetic: a laboratory manual and handbook for *Escherichia coli* and related bacteria; Laboratories CSH, editor: Cold Spring Habor, New York.

[37] Nagel G, Lahrz A, Dersch P (2001) Environmental control of invasin expression in *Yersinia pseudotuberculosis* is mediated by regulation of RovA, a transcriptional activator of the SlyA/Hor family. Mol Microbiol 41: 1249–1269. 11580832 10.1046/j.1365-2958.2001.02522.x

[38] Schaake J, Kronshage M, Uliczka F, Rohde M, Knuuti T, et al (2013) Human and animal isolates of *Yersinia enterocolitica* show significant serotype-specific colonization and host-specific immune defense properties. Infection and Immunity 81: 4013–4025. 23959720 10.1128/IAI.00572-13 PMC3811832

[39] Uliczka F, Pisano F, Kochut A, Opitz W, Herbst K, et al (2011) Monitoring of gene expression in bacteria during infections using an adaptable set of bioluminescent, fluorescent and colorigenic fusion vectors. PLoS One 6: e20425. 21673990 10.1371/journal.pone.0020425 PMC3108616

[40] Pisano F, Kochut A, Uliczka F, Geyer R, Stolz T, et al (2012) *In vivo*-induced InvA-like autotransporters Ifp and InvC of *Yersinia pseudotuberculosis* promote interactions with intestinal epithelial cells and contribute to virulence. Infect Immun 80: 1050–1064. 22158741 10.1128/IAI.05715-11 PMC3294637

[41] Hanski C, Naumann M, Grutzkau A, Pluschke G, Friedrich B, et al (1991) Humoral and cellular defense against intestinal murine infection with *Yersinia enterocolitica* . Infection and Immunity 59: 1106–1111. 1997413 10.1128/iai.59.3.1106-1111.1991 PMC258374

[42] Autenrieth IB, Hantschmann P, Heymer B, Heesemann J (1993) Immunohistological characterization of the cellular immune response against *Yersinia enterocolitica* in mice: evidence for the involvement of T lymphocytes. Immunobiology 187: 1–16. 8505058 10.1016/S0171-2985(11)80241-X

[43] Autenrieth IB, Tingle A, Reske-Kunz A, Heesemann J (1992) T lymphocytes mediate protection against *Yersinia enterocolitica* in mice: characterization of murine T-cell clones specific for *Y. enterocolitica* . Infect Immun 60: 1140–1149. 1541529 10.1128/iai.60.3.1140-1149.1992 PMC257605

[44] Bergman MA, Loomis WP, Mecsas J, Starnbach MN, Isberg RR (2009) CD8(+) T cells restrict *Yersinia pseudotuberculosis* infection: bypass of anti-phagocytosis by targeting antigen-presenting cells. PLoS Pathog 5: e1000573. 19730693 10.1371/journal.ppat.1000573 PMC2731216

[45] Kumar S, Balakrishna K, Agarwal GS, Merwyn S, Rai GP, et al (2009) Th1-type immune response to infection by pYV-cured *phoP-phoQ* null mutant of *Yersinia pseudotuberculosis* is defective in mouse model. Antonie Van Leeuwenhoek 95: 91–100. 18985430 10.1007/s10482-008-9292-5

[46] Svanborg C, Godaly G, Hedlund M (1999) Cytokine responses during mucosal infections: role in disease pathogenesis and host defence. Current Opinion in Microbiology 2: 99–105. 10047563 10.1016/s1369-5274(99)80017-4

[47] Chain PS, Carniel E, Larimer FW, Lamerdin J, Stoutland PO, et al (2004) Insights into the evolution of *Yersinia pestis* through whole-genome comparison with *Yersinia pseudotuberculosis* . Proc Natl Acad Sci U S A 101: 13826–13831. 15358858 10.1073/pnas.0404012101 PMC518763

[48] Bolin I, Norlander I, Wolf-Watz H (1982) Temperature-inducible outer membrane protein of *Yersinia pseudotuberculosis* and *Yersinia enterocolitica* is associated with the virulence plasmid. Infect Immun 37: 506–512. 6749681 10.1128/iai.37.2.506-512.1982 PMC347563

[49] Lockman HA, Gillespie RA, Baker BD, Shakhnovich E (2002) *Yersinia pseudotuberculosis* produces a cytotoxic necrotizing factor. Infect Immun 70: 2708–2714. 11953417 10.1128/IAI.70.5.2708-2714.2002 PMC127951

[50] Sun YC, Koumoutsi A, Darby C (2009) The response regulator PhoP negatively regulates *Yersinia pseudotuberculosis* and *Yersinia pestis* biofilms. FEMS Microbiol Lett 290: 85–90. 19025559 10.1111/j.1574-6968.2008.01409.x PMC2610865

[51] Balada-Llasat JM, Mecsas J (2006) Yersinia has a tropism for B and T cell zones of lymph nodes that is independent of the type III secretion system. PLoS Pathog 2: e86. 16948531 10.1371/journal.ppat.0020086 PMC1557584

[52] Paczosa MK, Fisher ML, Maldonado-Arocho FJ, Mecsas J (2013) *Yersinia pseudotuberculosis* uses Ail and YadA to circumvent neutrophils by directing Yop translocation during lung infection. Cellular Microbiology. 10.1111/cmi.12219 PMC3981955 24119087

[53] Simonet M, Falkow S (1992) Invasin expression in *Yersinia pseudotuberculosis* . Infect Immun 60: 4414–4417. 1398952 10.1128/iai.60.10.4414-4417.1992 PMC257481

[54] Maldonado-Arocho FJ, Green C, Fisher ML, Paczosa MK, Mecsas J (2013) Adhesins and host serum factors drive Yop translocation by yersinia into professional phagocytes during animal infection. PLoS Pathog 9: e1003415. 23818844 10.1371/journal.ppat.1003415 PMC3688556

[55] Heise T, Dersch P (2006) Identification of a domain in *Yersinia* virulence factor YadA that is crucial for extracellular matrix-specific cell adhesion and uptake. Proc Natl Acad Sci U S A 103: 3375–3380. 16488979 10.1073/pnas.0507749103 PMC1413876

[56] Bliska JB, Wang X, Viboud GI, Brodsky IE (2013) Modulation of innate immune responses by *Yersinia* type III secretion system translocators and effectors. Cellular Microbiology 15: 1622–1631. 23834311 10.1111/cmi.12164 PMC3788085

[57] Palonen E, Kangas S, Somervuo P, Lindstrom M, Fredriksson-Ahomaa M, et al (2013) Sequencing of virulence genes shows limited genetic variability in *Yersinia pseudotuberculosis* . Foodborne Pathog Dis 10: 21–27. 23153288 10.1089/fpd.2012.1247

[58] Laukkanen-Ninios R, Didelot X, Jolley KA, Morelli G, Sangal V, et al (2011) Population structure of the *Yersinia pseudotuberculosis* complex according to multilocus sequence typing. Environ Microbiol 13: 3114–3127. 21951486 10.1111/j.1462-2920.2011.02588.x PMC3988354

[59] Anand RD, Sertil O, Lowry CV (2004) Restriction digestion monitors facilitate plasmid construction and PCR cloning. BioTechniques 36: 982–985. 15211749 10.2144/04366ST03

