# Supplementary figures and images for: Influence of PhoP and Intra-Species Variations on Virulence of Yersinia pseudotuberculosis during the Natural Oral Infection Route
Source: PLoS One. 2014 Jul 30;9(7):e103541. doi: 10.1371/journal.pone.0103541 (PMC4116203; doi:10.1371/journal.pone.0103541)

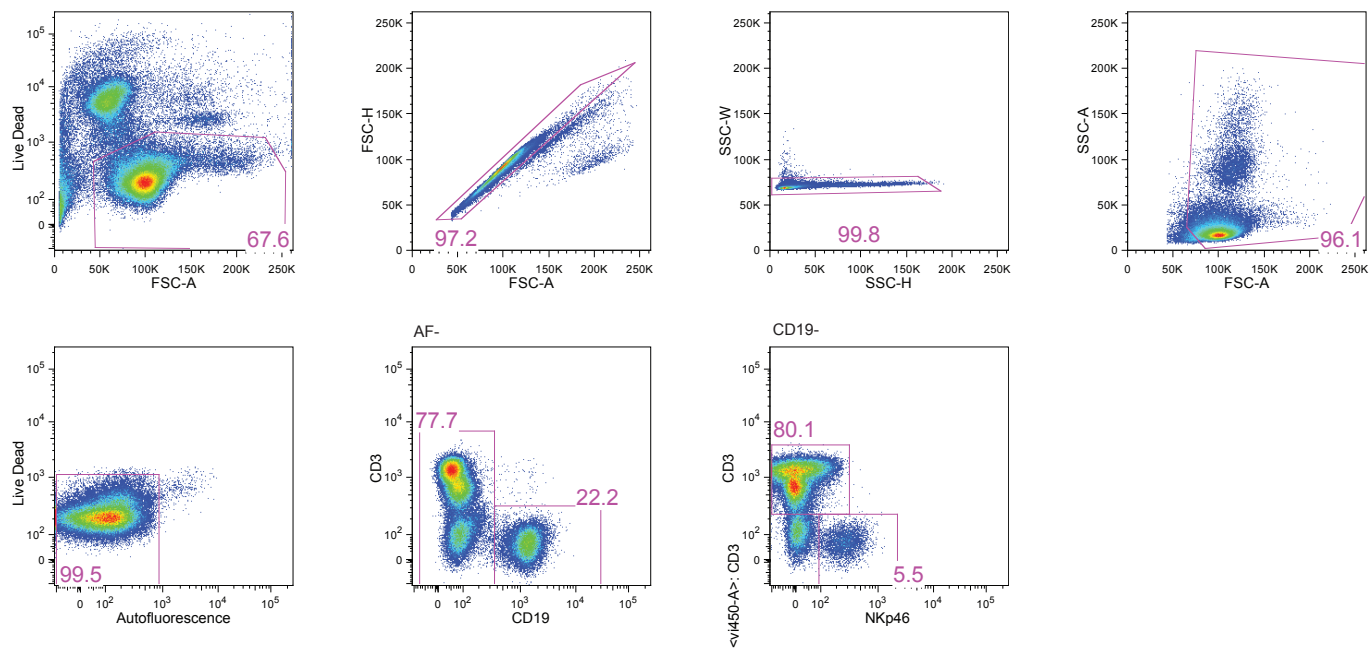

**Fig. S1** Pisano *et al.* 2014

Supplement: Figure S1 — Gating strategy for T cells, B cells and NK cells. The collected flow cytometry data was compensated and analyzed with FlowJo. At first, cells were gated for living cells by plotting Live Dead versus FSC-A followed by a double doublet-exclusion (FSC-A against FSC-H; SSC-H against SSC-W). The remaining singlets were gated for leukocytes by plotting FSC-A against SSC-A. At next, the auto fluorescent cells (AF+) were excluded (auto-fluorescence (AF) versus Live Dead). The remaining autofluorescence negative (AF−) leukocytes were gated for CD19+ cells (B cells). Subsequently, the CD19− cells were gated for CD3+ cells (T cells) and NKp46+ (NK cells). Cell proportions of living T cells, B cells and NK cells were calculated by the frequency of living cells. (PDF) [file pone.0103541.s001.pdf]

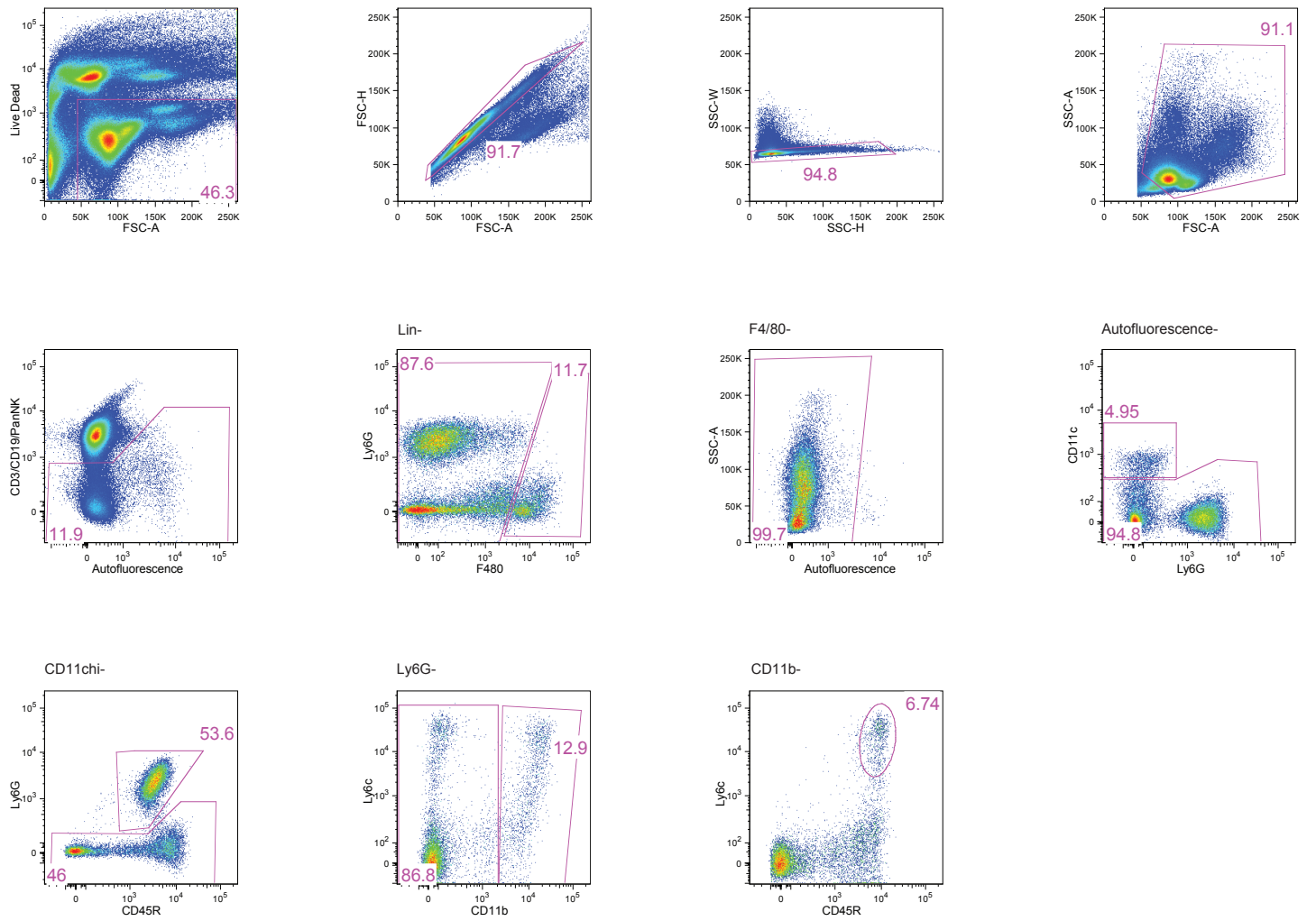

**Fig. S2** Pisano *et al.* 2014

Supplement: Figure S2 — Gating strategy for cDCs, pDCs, neutrophils, macrophages and monocytes. The collected flow cytometry data was compensated and analyzed with FlowJo. At first, cells were gated for living cells by plotting Live Dead versus FSC-A followed by a double doublet-exclusion (FSC-A against FSC-H; SSC-H against SSC-W). The remaining singlets were gated for leukocytes by plotting FCS-A against SSC-A. At next, the leukocytes were gated for CD3/CD19/panNK lineage negative cells (Lin−) by plotting AF against CD3/CD19/panNK. The Lin− cells were gated for F4/80hi cells (macrophages) by plotting F4/80 against Ly6G. Subsequently, the F4/80hi negative cells were gated against auto-fluorescence and SSC-A to exclude AF+ cells. Afterwards, the AF− cells were gated for CD11chi cells (cDCs) by plotting Ly6G against CD11c. The CD11chi negative cells were gated against CD45R and Ly6G for Ly6G+ cells (neutrophils). There after, the Ly6G− cells were plotted against CD11b and Ly6C for CD11b+ cells (monocytes). Finally, The CD11b− cells were gated for CD45R+Ly6C+ cells (pDCs). Cell proportions of living macrophages, cDCs, neutrophils, monocytes and pDCs were calculated by the frequency of living cells. (PDF) [file pone.0103541.s002.pdf]

# Peyer's Patches

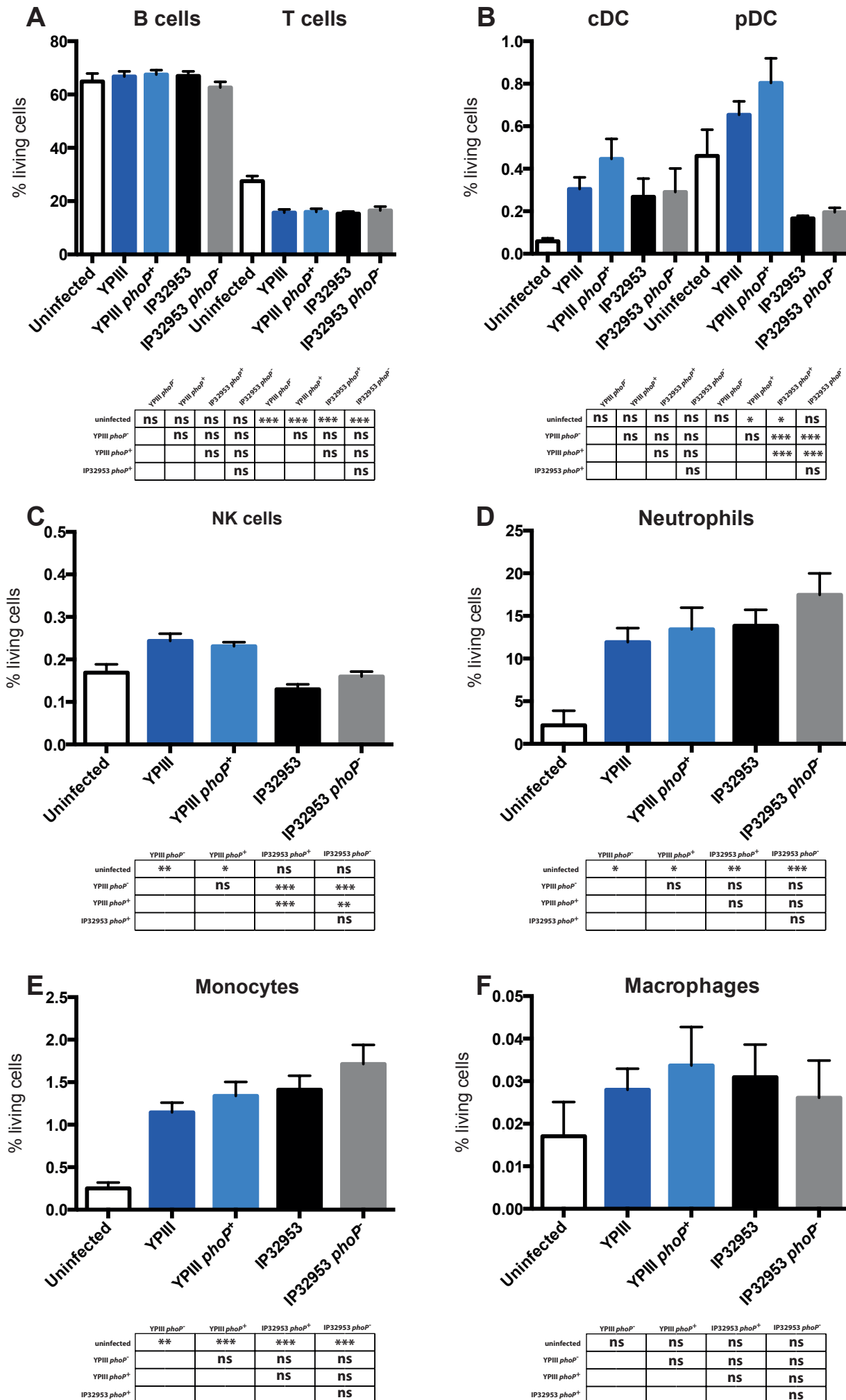

Fig. S3 Pisano et al. 2014

Supplement: Figure S3 — Immune response analysis in the PPs of BALB/c mice induced by phoP + or phoP − derivates of Y. pseudotuberculosis YPIII or IP32953. Mice were challenged with 2×10^8^ CFU of Y. pseudotuberculosis strains YPIII (phoP −) (n = 20), YP149 (YPIII phoP +) (n = 20), IP32953 (phoP +) (n = 20) and YPIP06 (IP32953 phoP −) (n = 20). A control group of uninfected mice (n = 15) was included. At day three postinfection proportions of living B cells and T cells (A), cDCs and pDCs (B), NK cells (C), neutrophils (D), monocytes (E), macrophages (F) in the PPs were analyzed. Data from four independent experiments were pooled. Population percentages were analyzed using One-way ANOVA with Tukey’s post hoc test (*, p<0.05; **, p<0.01; ***, p<0.001). (PDF) [file pone.0103541.s003.pdf]

# Mesenteric lymph nodes

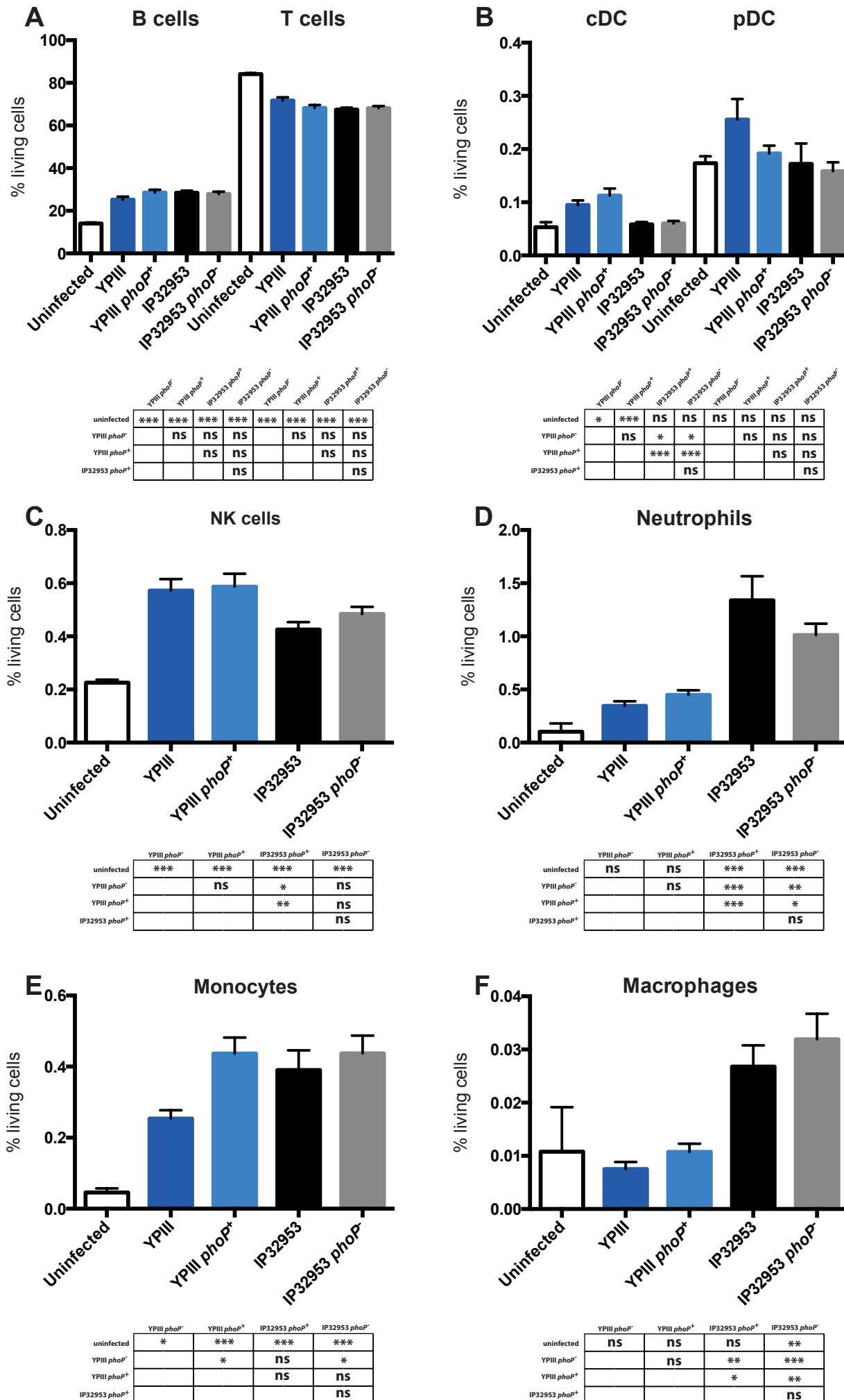

Fig. S4 Pisano et al. 2014

Supplement: Figure S4 — Immune response analysis in the MLNs of BALB/c mice induced by phoP + or phoP − derivates of Y. pseudotuberculosis YPIII or IP32953. Mice were challenged with 2×10^8^ CFU of Y. pseudotuberculosis strains YPIII (phoP −) (n = 20), YP149 (YPIII phoP +) (n = 20), IP32953 (phoP +) (n = 20) and YPIP06 (IP32953 phoP −) (n = 20). A control group of uninfected mice (n = 15) was included. At day three postinfection proportions of living B cells and T cells (A), cDCs and pDCs (B), NK cells (C), neutrophils (D), monocytes (E), macrophages (F) in the MLNs were analyzed. Data from four independent experiments were pooled. Population percentages were analyzed using One-way ANOVA with Tukey’s post hoc test (*, p<0.05; **, p<0.01; ***, p<0.001). (PDF) [file pone.0103541.s004.pdf]

## Spleen

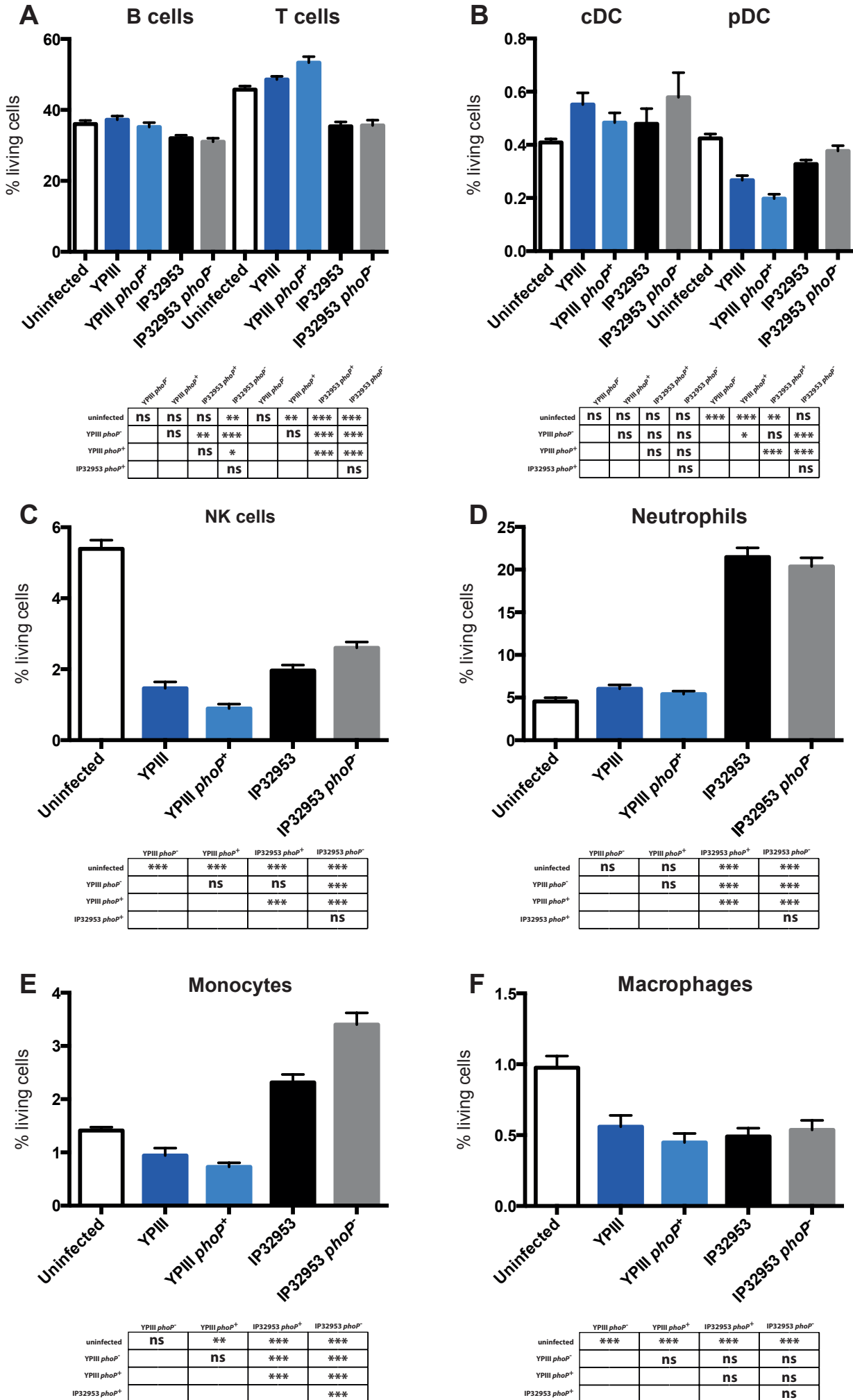

**Fig. S5** Pisano *et al.* 2014

Supplement: Figure S5 — Immune response analysis in the spleen of BALB/c mice induced by phoP + or phoP − derivates of Y. pseudotuberculosis YPIII or IP32953. Mice were challenged with 2×10^8^ CFU of Y. pseudotuberculosis strains YPIII (phoP −) (n = 20), YP149 (YPIII phoP +) (n = 20), IP32953 (phoP +) (n = 20) and YPIP06 (IP32953 phoP −) (n = 20). A control group of uninfected mice (n = 15) was included. At day three postinfection proportions of living B cells and T cells (A), cDCs and pDCs (B), NK cells (C), neutrophils (D), monocytes (E), macrophages (F) in the spleen were analyzed. Data from four independent experiments were pooled. Population percentages were analyzed using One-way ANOVA with Tukey’s post hoc test (*, p<0.05; **, p<0.01; ***, p<0.001). (PDF) [file pone.0103541.s005.pdf]

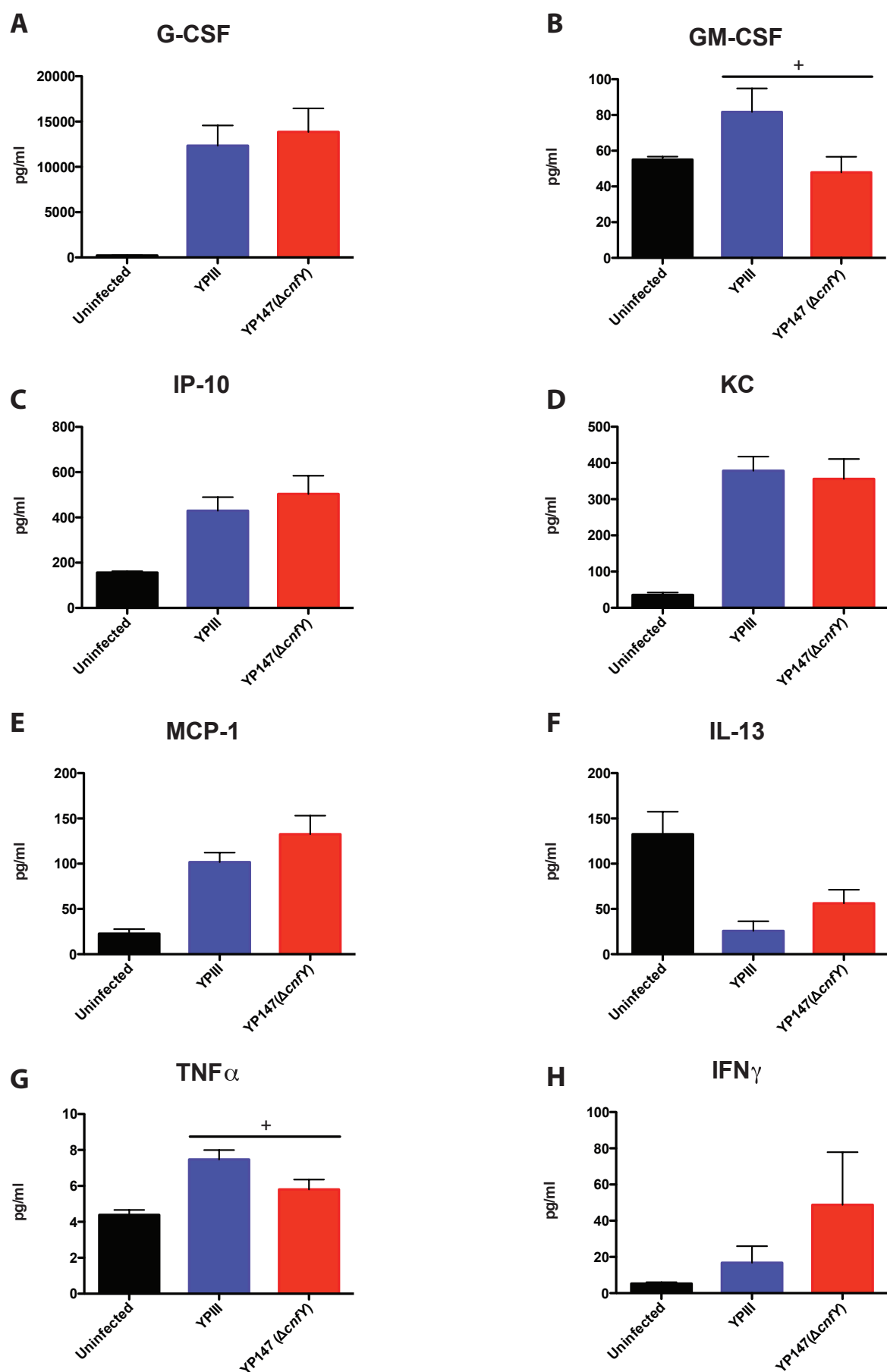

**Fig. S6** Pisano *et al.* 2014

Supplement: Figure S6 — Cytokine analysis of the serum of BALB/c mice infected with Y. pseudotuberculosis YPIII or YP147. Mice were challenged with 2×10^8^ CFU of Y. pseudotuberculosis strains YPIII (phoP −) (n = 8) or YP147 (YPIII phoP − ΔcnfY) (n = 8); a control group of uninfected mice (n = 5) was included. At day three postinfection blood was isolated to determine the cytokine profile of the serum. G-CSF (A), GM-CSF (B), IP-10 (C), KC (D), MCP-1 (E), IL-13 (F), TNFα (G), IFNγ (H). Cytokine levels were statistically analysed using One-way ANOVA with Tukey’s post hoc test (+, p<0.05; ++, p<0.01; +++, p<0.001). + indicate a comparison to the uninfected group. (PDF) [file pone.0103541.s006.pdf]
